# X-chromosome inactivation patterns depend on age and tissue but not conception method in humans

**DOI:** 10.1007/s10577-023-09717-9

**Published:** 2023-01-25

**Authors:** Patrycja Juchniewicz, Anna Kloska, Karolina Portalska, Joanna Jakóbkiewicz-Banecka, Grzegorz Węgrzyn, Joanna Liss, Piotr Głodek, Stefan Tukaj, Ewa Piotrowska

**Affiliations:** 1 Department of Medical Biology and Genetics, Faculty of Biology, University of Gdańsk, Wita Stwosza 59, 80-308 Gdańsk, Poland; 2 Department of Molecular Biology, Faculty of Biology, University of Gdańsk, Wita Stwosza 59, 80-308 Gdańsk, Poland; 3 Research and Development Center, INVICTA, Sopot, Poland

**Keywords:** X-chromosome inactivation, XCI, Dosage compensation, Human development, Placenta, Methods of conception

## Abstract

Female somatic X-chromosome inactivation (XCI) balances the X-linked transcriptional dosages between the sexes, randomly silencing the maternal or paternal X chromosome in each cell of 46,XX females. Skewed XCI toward one parental X has been observed in association with ageing and in some female carriers of X-linked diseases. To address the problem of non-random XCI, we quantified the XCI skew in different biological samples of naturally conceived females of different age groups and girls conceived after in vitro fertilization (IVF). Generally, XCI skew differed between saliva, blood, and buccal swabs, while saliva and blood had the most similar XCI patterns in individual females. XCI skew increased with age in saliva, but not in other tissues. We showed no significant differences in the XCI patterns in tissues of naturally conceived and IVF females. The gene expression profile of the placenta and umbilical cord blood was determined depending on the XCI pattern. The increased XCI skewing in the placental tissue was associated with the differential expression of several genes out of 40 considered herein. Notably, skewed XCI patterns (> 80:20) were identified with significantly increased expression levels of four genes: *CD44*, *KDM6A*, *PHLDA2*, and *ZRSR2*. The differences in gene expression patterns between samples with random and non-random XCI may shed new light on factors contributing to the XCI pattern outcome and indicate new paths in future research on the phenomenon of XCI skewing.

**Supplementary Information:**

The online version contains supplementary material available at 10.1007/s10577-023-09717-9.

## Introduction

X-chromosome inactivation (XCI) is an epigenetically regulated mechanism of dosage compensation balancing expression levels of X-linked genes between females (XX) and males (XY). In this process, either maternally or paternally inherited X chromosome present in individual cells of the developing female embryo is marked for inactivation. It means that several hundred physically linked loci become concomitantly and stably silenced (Patrat et al. 2020); this silencing is inherited through subsequent somatic cell divisions. Still, up to one-third of human X-chromosomal genes escape this process and are expressed from both the active and inactive X chromosomes in female cells (Tukiainen et al. 2017). The degree of XCI incompleteness varies between genes, tissues, and individuals (Shvetsova et al. 2019), and remains poorly characterized.

Sixty years after Mary Lyon put forth the hypothesis of random X-chromosome inactivation (Lyon 1961), skewed XCI has been reported to associate with many rare and common disorders (Plenge et al. 2002; Migeon 2020; Juchniewicz et al. 2021). As for the general female population, the literature is much scarcer. The most comprehensive population studies of healthy women and girls showed XCI skewing ratio > 70:30 in 25–27% of the analysed females, and a ratio > 80:20 in 8–10%, while the ratio > 90:10 was identified only in 1.8% (Amos-Landgraf et al. 2006; Shvetsova et al. 2019). However, when the threshold for a skewed XCI ratio was defined as ≥ 65:35, nearly 50% of the population demonstrated imbalanced inactivation of the two X chromosomes (Shvetsova et al. 2019).

Besides the subjectively defined threshold for preferential inactivation of either parental chromosome, the pattern of XCI (random or skewed) depends on the age of a woman and the type of examined tissues (Sharp et al. 2000; Amos-Landgraf et al. 2006; Ørstavik 2009). The randomness of XCI in different tissues may range from 50:50 to 80:20 in the same individual which can be attributed to the fact that cells with high mitotic activity have a more skewed pattern than cells with lower mitotic activity (Sharp et al. 2000; Knudsen et al. 2007; Bolduc et al. 2008). Still, most studies on XCI have been performed in DNA from peripheral blood with intensely dividing cells (Ørstavik 2009). When XCI patterns were determined in easily accessible tissues (buccal epithelium, blood, and hair follicle) and compared with XCI patterns in several inaccessible tissues (heart, thyroid, kidney, liver, muscle, and ovary), it appeared that buccal epithelium was preferable over peripheral blood cells for predicting XCI pattern in inaccessible tissues. The ovary was the only inaccessible tissue showing a poor correlation to the buccal epithelium and blood cells but had a good correlation to hair follicles instead (de Hoon et al. 2015).

The frequency of XCI skewing was suggested to increase with age, especially over 55 years (Kristiansen et al. 2005). Longitudinal studies of XCI performed for the same females at an average interval of two decades confirmed a significant difference in the degree of XCI skewing with time in females who were 60 years or older at the time of the first sampling (Sandovici et al. 2004). On the other hand, Shvetsova et al. (2019) did not observe an increase in skewing with age while studying the population with the age range of 20–64 years. If the age-related skewing of XCI is more prevalent in the general population, its biological significance remains unclear, but one of its possible consequences might be the manifestation of X-linked disorders in elderly females (Cazzola et al. 2000; Au et al. 2004).

XCI occurs in early embryonic life, but the exact timing in humans is still elusive (Goto and Monk 1998; Patrat et al. 2020). The current knowledge of human XCI is mainly based on the studies performed on embryos obtained by in vitro fertilization (IVF) or pluripotent stem cells.

Epidemiological data indicate that children conceived in vitro have a greater relative risk of low birth weight, major and minor birth defects, and rare disorders involving imprinted genes, suggesting that epigenetic changes may be associated with assisted reproduction (Katari et al. 2009). Key events in epigenetic reprogramming occur in mammals during germ cell development and early embryogenesis. IVF involves the manipulation of many steps of conception from the stimulation of gamete production to the ex vivo culture of embryos and includes risk factors such as advanced maternal age, sperm aneuploidy, superovulation, in vitro handling of egg and sperm, and embryo growth in culture. These factors can lead to epigenetic alterations, slow embryo growth, or cause cell selection resulting in skewed XCI (Wu et al. 2014). Studies on mice have shown that superovulation disrupts the acquisition of imprints in growing oocytes and maternal-effect gene products required for imprint maintenance during preimplantation development (Market-Velker et al. 2010). Moreover, a skewed sex ratio in mouse offspring was shown to result from impaired XCI following the IVF procedure (Tan et al. 2016).

Determination of the effects of IVF on epigenetic processes in humans, such as the XCI event, is the current topic of our interest. Thus, this study aimed to compare XCI patterns between easily accessible biological materials (saliva, buccal swab, and blood) of females of different age groups. To elucidate the effect of assisted reproductive technologies on the human XCI process, we compared the XCI patterns of girls born after assisted versus natural conception. To better characterize how the XCI process is governed in embryonic development, we also examined the status of XCI in placental tissue and umbilical cord blood and determined gene expression profiles of samples with different XCI patterns.

## Materials and methods

### Study population and sample collection

Samples of saliva, blood, or buccal swabs were collected from 227 naturally conceived females of three age groups: 0–12, 13–35, and over 35 years for genomic DNA extraction and XCI pattern analysis. Each participant or her parent received a package containing three sample collection kits: (i) FTA classic card with multi-barrier pouch (Whatman, UK) and a single-use safety lancet Medlance (HTL-STREFA, Poland) for dry blood spot collection; (ii) two sterile swabs in tubes with a specimen bag (Hagmed, Poland) for buccal swab collection; and (iii) Oragene DNA self-collection kit: OG-500 or OG-575 (DNA Genotek, Canada) for saliva sampling. The kits included step-by-step instructions for the self-collection of biospecimens. All samples were coded and handled as anonymous.

Similar sample collection packages were given to parents of 38 girls (including four pairs of female twins) conceived in vitro in the age range 0–5 years. To compare XCI patterns in girls conceived by IVF and naturally, samples from a commensurate group of girls from the youngest group of naturally conceived females were selected. Table 1 shows the characteristics of these two groups as well as three age groups of naturally conceived females and the numbers of obtained samples.

**Table 1:** Characteristics of the groups compared in this study

Groups	*N*	Age range (years)	Mean age (years)	Number of samples			
				Saliva	Blood	Buccal swab	Complete set^a^
Age groups of naturally conceived females							
0–12 years	66	0–12	5.9	54	45	50	34
13–35 years	89	15–35	25.9	87	49	53	48
> 35 years	72	36–84	47.9	71	43	44	42
Groups of girls conceived by IVF and naturally							
IVF	38	0–5	1.89	21	14	34	12
NC	37	0–6	2.73	26	24	26	14

^a^Complete set of saliva, blood, and buccal swab; *IVF*, conceived by in vitro fertilization (in all cases the oocytes were inseminated by intracytoplasmic sperm injection, ICSI); *NC*, naturally conceived

Samples of umbilical cord blood and placental tissue were collected from nine female neonates born naturally at full term after single pregnancy as sources of DNA and RNA. Umbilical cord blood for DNA isolation was collected into tubes with EDTA and for RNA isolation into PAXgene® blood RNA tubes (PreAnalytiX, Switzerland) and stored at – 20 °C. Placental tissue samples were collected within 30 min after delivery of the placenta. The biopsies (2.0–2.5 cm^3^) were taken from four different areas of the placenta from the foetal side and rinsed extensively in phosphate-buffered saline to remove blood. Each sample was divided into two halves: one for RNA and the other for DNA isolation. The first half was placed in RNAlater (Sigma Aldrich, USA), incubated at 4 °C overnight, then removed from RNAlater, and transferred to a fresh tube for storage at − 80 °C according to the guidelines provided by the RNAlater manufacturer. The second half designated for DNA extraction was placed in an empty tube and stored at – 20 °C. Additionally, paired saliva samples from mothers of newborns were collected for DNA testing.

### Nucleic acid extraction

The QIAamp DNA Mini kit (Qiagen, USA) was used to extract genomic DNA from buccal swabs, dried blood spots, umbilical cord blood, and placenta, and the prepIT®•L2P reagent (DNA Genotek, Canada) was used to isolate DNA from saliva samples. RNA from umbilical cord blood was extracted using PAXgene® blood RNA kit (PreAnalytiX, Switzerland) and from the placenta—using RNeasy® micro kit (Qiagen, USA).

### X-chromosome inactivation analysis

The methylation-sensitive HUMARA (human androgen receptor) assay (Allen et al. 1992) with previously described modifications (Juchniewicz et al. 2018) was used to determine XCI patterns in all DNA samples. If the androgen receptor locus was uninformative, X-inactivation status was assessed at the *DXS6673E* locus using primers designed by Beever et al. (Beever et al. 2003) and their PCR protocol with the following modifications: both digested and undigested samples (50 ng) were amplified using 1 × PCR reaction buffer with 1.5 mM MgCl_2_, 200 μM of each dNTP, 0.2 μM of each primer (the forward PCR primer was labelled with the 6-FAM), and 1 U of Taq DNA polymerase (Roche Applied Science, USA) in a total volume of 15 μl. The PCR conditions were 95 °C for 5 min (initial denaturation) followed by 35 cycles of 95 °C for 40 s, 58 °C for 40 s, and 72 °C for 40 s, with a final extension at 72 °C for 7 min. PCR products were separated by capillary electrophoresis on an ABI Prism 310 genetic analyser (Applied Biosystems, USA) in denaturing conditions. Product length and peak areas were analysed using Peak Scanner Software v1.0 (Applied Biosystems, USA). All samples were analysed in duplicate, and the ratios obtained were averaged. DNA of a female with 80:20 skewed XCI was used as a control in each assay run to assess inter-assay variability. The XCI pattern was classified as random (50:50 to 80:20), skewed (80:20 to 90:10) or extremely skewed (90:10 and more).

### Real-time quantitative RT-PCR

Based on the literature search, 40 growth-related imprinted (Nelissen et al. 2014; Kappil et al. 2015; Kleijkers et al. 2015; Litzky et al. 2017) or XCI-escaping (Tukiainen et al. 2017) genes were selected for placental and umbilical blood gene expression profiling (*ABCA1*, *ACE2*, *AR*, *BID*, *BLCAP*, *CD44*, *CD99*, *CDKN1C*, *DLK1*, *ERP27*, *GRB10*, *GYG2*, *H19*, *HECW2*, *IGF2*, *IL9R*, *JPX*, *KDM5C*, *KDM6A*, *MEG3*, *MEST*, *NAP1L5*, *NDN*, *NNAT*, *OCA2*, *OTC*, *P2RY8*, *PAX8*, *PHLDA2*, *PLAGL1*, *PLCXD1*, *PNPLA4*, *PRKX*, *RPS4X*, *STS*, *SULT4A1*, *TUBGCP5*, *XIST*, *ZFX*, *ZRSR2*). Total RNA was reverse-transcribed into cDNA using transcriptor first-strand cDNA synthesis kit. Real-time qRT-PCR was performed using real-time ready custom panel and LightCycler 480 Probes Master on a LightCycler® 480 System platform (all from Roche Applied Science, Germany). Standard protocols as per manufacturer recommendations were followed.

Three housekeeping genes—*SDHA*, *YWHAZ*, and *TBP*—were analysed alongside the genes of interest using the same procedure. Expression stability of housekeeping genes was determined from raw cycle threshold (*C*_*t*_) values using the BestKeeper tool (Pfaffl et al. 2004); *SDHA* and *YWHAZ* were selected as the most stable and applied as reference genes for normalization of results obtained for genes of interest. Relative expression (RE) was calculated with the 2^ΔCt^ method (Livak and Schmittgen 2001), where Δ*C*_*t*_ was obtained by subtracting the *C*_*t*_ of the gene of interest from the geometric mean of *C*_*t*_ of two reference genes. To calculate the gene expression fold change (FC), RE for each sample was normalized to the mean RE obtained for samples of the group with XCI of 50–59%. Fold change values were log_2_-transformed before statistical analyses and visualization.

### Statistical analysis

Pearson’s *χ*^2^ test was used to compare frequencies of XCI ratios in each 10% increment from 50 to 100% as well as random/skewed XCI in different tissues in each age group, and Kolmogorov–Smirnov test was performed to compare distributions. Kruskal–Wallis test followed by Dunn’s multiple comparison post hoc test was used to compare medians of XCI ratio in different biological samples of three different age groups. Pearson’s correlation coefficient (*r*) was calculated to determine the association between X-inactivation differences in samples of individual females and their ages. The *χ*^2^ test for independence (two-way) was used to determine dependencies between variables. The *t*-test was used to evaluate significant changes in gene expression in comparison to samples with the XCI of 50–59%, and the Benjamini–Hochberg procedure was used for controlling the false discovery rate; significance was declared if the Benjamini–Hochberg adjusted *P* value was smaller than the false discovery rate set at 0.1. One-way ANOVA with Fisher’s least significant difference post hoc analysis was used to determine the differences in mean gene expression among all XCI skewing groups; significance was considered at *P* value < 0.05. Analyses were performed using Statistica v.13 (TIBCO Software Inc., USA).

### Data visualization

Venn diagrams were constructed using the InteractiVenn tool (Heberle et al. 2015). Volcano plots were generated using VolcaNoseR (Goedhart and Luijsterburg 2020) with log_2_(FC) ≥|1.0| set as a fold change threshold and − log10(adjusted *P* value) ≥ 1.0 as the significance threshold. Heatmaps were created using Heatmapper (Babicki et al. 2016) software with Euclidean algorithm used for distance measurement between rows and columns and average linkage method used for computing hierarchical clustering. Bland–Altman plots (with the corresponding calculation of limits of agreement) were generated using the BA-plotteR tool (Goedhart and Rishniw 2021). Boxplots were created using the Boxplot v0.1.0 tool, and Uniform Manifold Approximation and Projection (UMAP) analysis was performed using the UMAP v0.1.0 tool—both available in Hiplot (https://hiplot.com.cn). Correlograms were generated with a web-based FaDA tool (Danger et al. 2021). Gene Set Annotation (GSAn) public web server was used for characterising gene lists with Gene Ontology (GO) terms (Ayllon-Benitez et al. 2020).

## Results

### X-chromosome inactivation patterns in blood, saliva, and buccal swabs of naturally conceived females

Considering paediatric development, age categories defined in this study followed age groupings suggested by the WHO (Knoppert et al. 2007). The group of females 0–12 years comprised neonates, infants, young children (< 6 years), and children (6–12 years). The age group 13–35 years covered adolescents and young adults, while the group > 35 years—middle-aged and older adults.

**Fig. 1:**
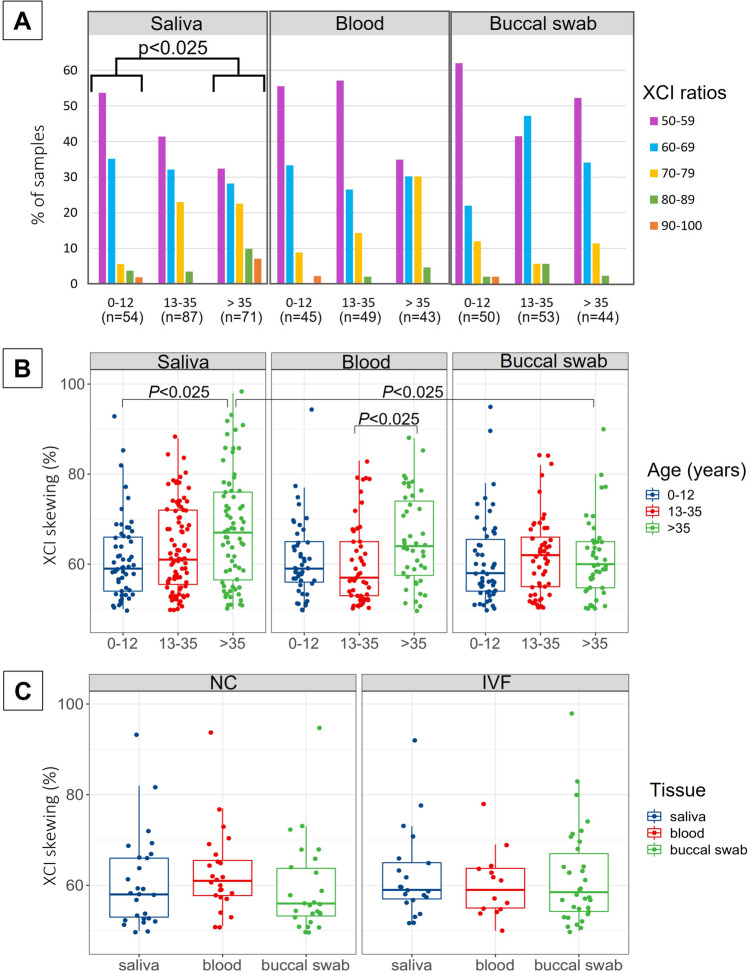
Distribution of XCI ratios in different biological samples from naturally conceived females in three different age groups. **A** XCI ratios expressed as ratios of the preferentially inactivated allele with a range of 50:50 to 100:0, with increments of 10%. Statistical significance in distribution (Kolmogorov–Smirnov test) is indicated. **B** XCI ratios expressed as continuous ratios of the preferentially inactivated allele with a range of 50:50 to 100:0. Statistical significance in the median (Kruskal–Wallis test with Dunn’s multiple comparison post hoc test) is indicated. **C** Distribution of XCI ratios in different biological samples from girls conceived naturally (NC) or by in vitro fertilization (IVF). XCI ratios are expressed as continuous ratios of the preferentially inactivated allele with a range of 50:50 to 100:0. Statistical significance was not observed (Kruskal–Wallis test). The box shows lower and upper quartiles and whiskers—minimum and maximum values of the data. The horizontal line inside the box represents median and dots—individual samples; the points located outside the whiskers represent outliers

No significant differences in the frequency of XCI ratios were found in blood and buccal swab samples from naturally conceived females of the three defined here age groups; however, the difference in the distribution of XCI ratios in saliva between 0 and 12 and > 35 years groups was statistically significant (Pearson’s χ^2^ test, *P* = 0.01; Kolmogorov–Smirnov test, *P* < 0.025). Additionally, the median XCI ratio in samples of saliva in groups 0–12 and > 35 years, blood in groups 13–35 and > 35, and saliva and buccal swabs in the group > 35 years differed significantly (Fig. 1).

Significantly increased frequency of skewed (80–90%) and extremely skewed (≥ 90%) XCI was observed only in the saliva samples obtained from the females of the oldest group, resulting in 10% and 7%, respectively (Pearson’s χ^2^ test,* P* = 0.036). The proportion of XCI skewing in other samples was not significant (Table 2).

**Table 2:** Distribution of XCI skewing in different biological samples from naturally conceived females of three different age groups

Biological sample	Age group (number of samples)	Number (%) of samples with random and skewed^a^ XCI pattern		
		Random	Skewed	Extremely skewed
Saliva	0–12 years (*n* = 54)	51 (94)	2 (4)	1 (2)
	13–35 years (*n* = 87)	83 (95)	4 (5)	0 (0)
	> 35 years (*n* = 71)	59 (83)	7 (10)*	5 (7)*
Blood	0–12 years (*n* = 45)	44 (98)	0 (0)	1 (2)
	13–35 years (*n* = 49)	48 (98)	1 (2)	0 (0)
	> 35 years (*n* = 43)	41 (95)	2 (5)	0 (0)
Buccal swab	0–12 years (*n* = 50)	48 (96)	1 (2)	1 (2)
	13–35 years (*n* = 53)	50 (94)	3 (6)	0 (0)
	> 35 years (*n* = 44)	43 (98)	1 (2)	0 (0)

^a^Skewed XCI means inactivation of the same allele in 80–90% and extremely skewed XCI in ≥ 90% of cells; *n*, number of samples; * indicates statistical significance (Pearson’s *χ*^2^ test, *P* < 0.05)

**Fig. 2:**
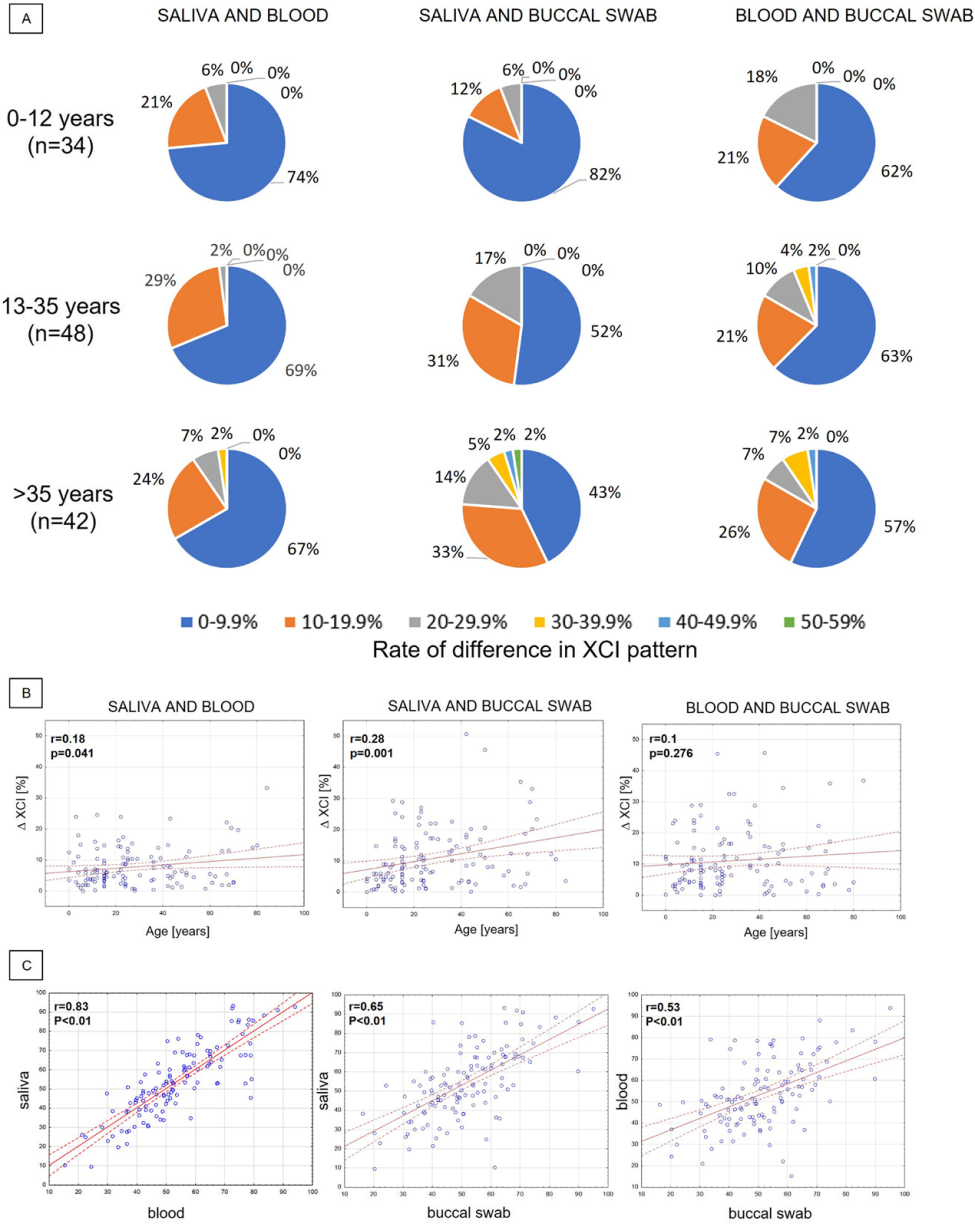
XCI patterns in pairs of samples (saliva–blood, saliva–buccal swab, and blood–buccal swab) in complete sets of saliva, blood, and buccal swab in 124 naturally conceived females. **A** Percentage of pairs of samples (saliva–blood, saliva–buccal swab, and blood–buccal swab) with XCI patterns differing by 0–9.9, 10–19.9, 20–29.9, 30–39.9, 40–49.9, and 50–59.9% within one person in three age groups. **B** Scatterplots of XCI differences in pairs of samples (saliva and blood, saliva and buccal swab, and blood and buccal swab) within one person versus age. **C** Correlation between XCI patterns of saliva and blood, saliva and buccal swab, and blood and buccal swab. *n*, number of complete sets of saliva, blood, and buccal swab; *r*, Pearson correlation coefficient

To check the variability in the sample-specific patterns of XCI within the same individual, the difference in XCI ratio in sample pairs in complete sets of saliva, blood, and the buccal swab was calculated. In the youngest group, the greatest difference (29.3%) was detected for the pair saliva (60:40) and buccal swab (90:10); the XCI ratio in blood in this girl was 61:39. In the group 13–35 years, the greatest difference (45.6%) was observed between blood (79:21) and buccal swab (34:66); saliva XCI in the set from this 22-year-old female was 55:45. In the oldest group, the highest difference of over 50% was found between saliva (11:89) and buccal swab (61:39); XCI ratio in the blood sample of this 42-year-old woman was 15:85. Besides the last two cases described above, there was one more woman (50 years old) with the difference of preferably inactivated X chromosome within the three collected samples. Her XCI patterns were 86:14, 75:25, and 40:60 in saliva, blood, and buccal swab, respectively. Figure 2A presents percentages of pairs of samples that differ in XCI patterns at different rates in our three age groups. XCI difference between saliva and buccal swab correlated weakly with age (*r* = 0.28, *P* = 0.001), a weaker correlation was observed for XCI difference between saliva and blood versus age (*r* = 0.18, *P* = 0.041), and no significant correlation was found between blood and buccal swab XCI difference and age (Fig. 2B). Saliva and blood had the most similar XCI patterns in individual females (*r* = 0.83, *P* < 0.01), while XCI patterns identified in blood and buccal swab had the weakest correlation (Fig. 2C).

### X-chromosome inactivation patterns in blood, saliva, and buccal swabs of females conceived by in vitro fertilization

Comparison of XCI pattern in IVF- and naturally conceived girls comprised females below 6 years, i.e. neonates, infants, and young children according to WHO guidelines (Knoppert et al. 2007). XCI patterns in the tested here biological samples did not differ significantly between girls conceived by IVF and naturally (Fig. 1C). In each group, there was one individual with extreme XCI skewing—in the IVF group, a 1-year-old girl had an XCI ratio of 92:8 in saliva and 98:2 in buccal swab (blood XCI in this girl was 78:22), and in naturally conceived group, one girl (5 years old) had XCI pattern of 93:7, 94:6, and 95:5 in saliva, blood, and buccal swab, respectively. In both cases, the preferential inactivation of the maternal X chromosome was detected.

### X-chromosome inactivation patterns in placental tissue and umbilical cord blood of females conceived naturally or by in vitro fertilization

XCI patterns were determined in the placental tissue and umbilical cord blood of nine girls—eight naturally and one IVF conceived. Table 3 displays neonate measurements of the girls together with the results of XCI analysis in the umbilical blood and placental samples as well as in the saliva of their mothers. Random XCI in all four examined placental samples was present in four subjects (501, 503, 504, 505), and in four others (502, 506, 519, 526), XCI was either random or skewed in samples taken from different areas of the same placenta, while in one subject (507), random, skewed, and extremely skewed XCI patterns were detected across four analysed placental samples. Two of our mother-daughter pairs shared the same alleles making the determination of X-chromosome parental origin not feasible. Of the remaining seven placentas, one showed the preferential inactivation of the maternally and one of the paternally inherited X, while five placentas showed heterogeneous XCI patterns with either maternally or paternally inherited X that was preferentially inactivated in the tested four samples. Correlation analysis of XCI skewing of nine mother-daughter pairs did not find a significant correlation (Pearson’s *r* =  − 0.3331, *P* = 0.3811) suggesting that the XCI status of the mother did not affect the XCI status of her daughter.

**Table 3:** Characteristics of neonates whose XCI patterns were analysed in umbilical blood and placental samples

Subject	Weight (g)	Length (cm)	Head circ. (cm)	Chest circ. (cm)	Apgar score^a^	Umbilical blood XCI^b^	Placenta XCI^b^	Mother’s age at delivery	Mother’s saliva XCI	Additional information
501	2560	51	31	31	10–10-10	65:35 (P)	55:45 (M) 60:40 (M) 69:31 (M) 76:24 (P)	27 years	62:38	NC
502	3170	55	34	33.5	10–10-10	54:46 (P)	53:47 (M) 54:46 (P) 59:41 (P) **81:19** (M)	35 years	**84:16**	NC
503	2950	52	33	32.5	10–09-08	61:39 (M)	55:45 (M) 57:43 (M) 66:34 (P) 79:21 (P)	25 years	78:22	NC
504	3800	57	33	34	10–10-10	59:41 (?)	55:45 (?) 58:42 (?) 74:26 (?) 76:24 (?)	26 years	61:39	NC, neonatal jaundice, Rh incompatibility, GBS( +)
505	3400	54	33	33	10–10-10	62:38 (P)	58:42 (P) 63:37 (P) 70:30 (P) 77:23 (P)	33 years	75:25	NC
506	3880	54	34	35	10–10-09	62:38 (?)	61:39 (?) 77:23 (?) **83:17** (?) **85:15** (?)	27 years	54:46	NC
507	3000	53	33	32.5	10–10-10	73:27 (M)	77:23 (P) 78:22 (P) **82:18** (P) **95:5** (M)	33 years	70:30	NC, congenital infection
519	3250	54	33	33	nd	64:36 (M)	63:37 (P) 79:21 (M) **81:19** (M) **84:16** (P)	29 years	53:47	IVF
526	3370	57	34	34	10–10-10	64:36 (M)	55:45 (M) 64:36 (M) 75:25 (M) **88:12** (M)	28 years	53:47	NC

^a^The score reported at 1, 5, and 10 min after birth; ^b^preferential inactivation of paternal (P) or maternal (M) X chromosome is indicated; (?)—the parental origin of preferentially inactivated X chromosome could not be determined; skewed and extremely skewed XCI patterns are indicated **in bold**; *circ*., circumference; *NC*, naturally conceived; *GBS(* +*)*, mother tested positive for group B Streptococcus; *IVF*, conceived by in vitro fertilization (the oocyte was inseminated by intracytoplasmic sperm injection, ICSI); *nd*, no data

**Fig. 3:**
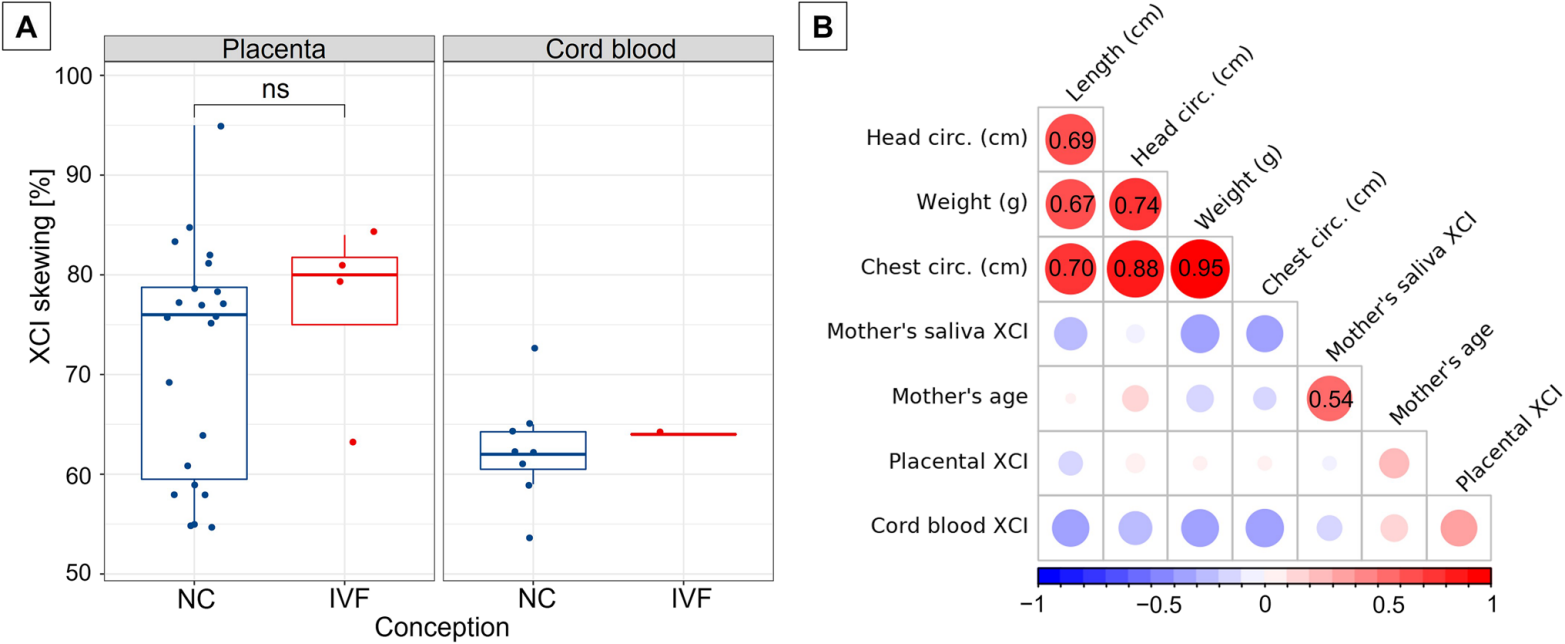
**A** XCI skewing in placental tissue and cord blood of females conceived naturally (NC) or by in vitro fertilization (IVF). XCI skewing is shown as the percentage of inactivation determined for the preferentially inactivated X chromosome. The box shows lower and upper quartiles and whiskers—minimum and maximum values of the data. The horizontal line inside the box represents median and dots—individual samples; the points located outside the box represent outliers. Group comparisons were performed with the *t*-test. **B** Correlation evidencing associations between neonate measurements, placental XCI, cord blood XCI, and mother’s saliva XCI and mother’s age. Pearson’s correlation coefficients (*r*) denoted as numbers on the correlogram were found statistically significant. circ., circumference; ns, not significant

The XCI ratios determined in placental tissue and umbilical cord blood were comparable between the naturally conceived and IVF groups (Fig. 3A); therefore, we decided to combine them and further analyse them together (especially since the IVF samples were withdrawn from only one individual). A significant positive correlation was observed between all neonate measurements (Fig. 3B). However, no significant correlations were found either among placental, cord blood, and mother’s saliva XCI or mother’s age, besides a moderate and significant correlation between the mother’s saliva XCI and age (Pearson’s *r* = 0.54, *P* = 0.004), (Fig. 3B).

### Gene expression profile of placental tissue and umbilical cord blood with different X-chromosome inactivation patterns

Placental tissue and umbilical cord blood samples were divided into XCI groups defined by 10% skewing intervals and analysed for gene expression with a panel of 40 genes using the real-time qRT-PCR technique (the expression of five genes, *IL9R*, *OTC*, *RPS4X*, *SULT4A1*, and *ZFX*, was undetectable; the other 35 genes are characterized in Supplemental Table S1). The *χ*^2^ test for independence showed that the XCI skewing variable in different placenta samples is independent of the individuum from whom it was collected (*χ*^2^ (24, *n* = 27) = 21.009, *P* < 0.05); thus, we pooled the placenta samples of different individuals and areas according to the XCI skewing value and not the individuum. The expression of 35 and 17 out of 40 profiled genes was detectable in placental tissue samples and umbilical blood, respectively. Differential gene expression was calculated relative to the expression assessed for samples with XCI of 50–59%.

**Fig. 4:**
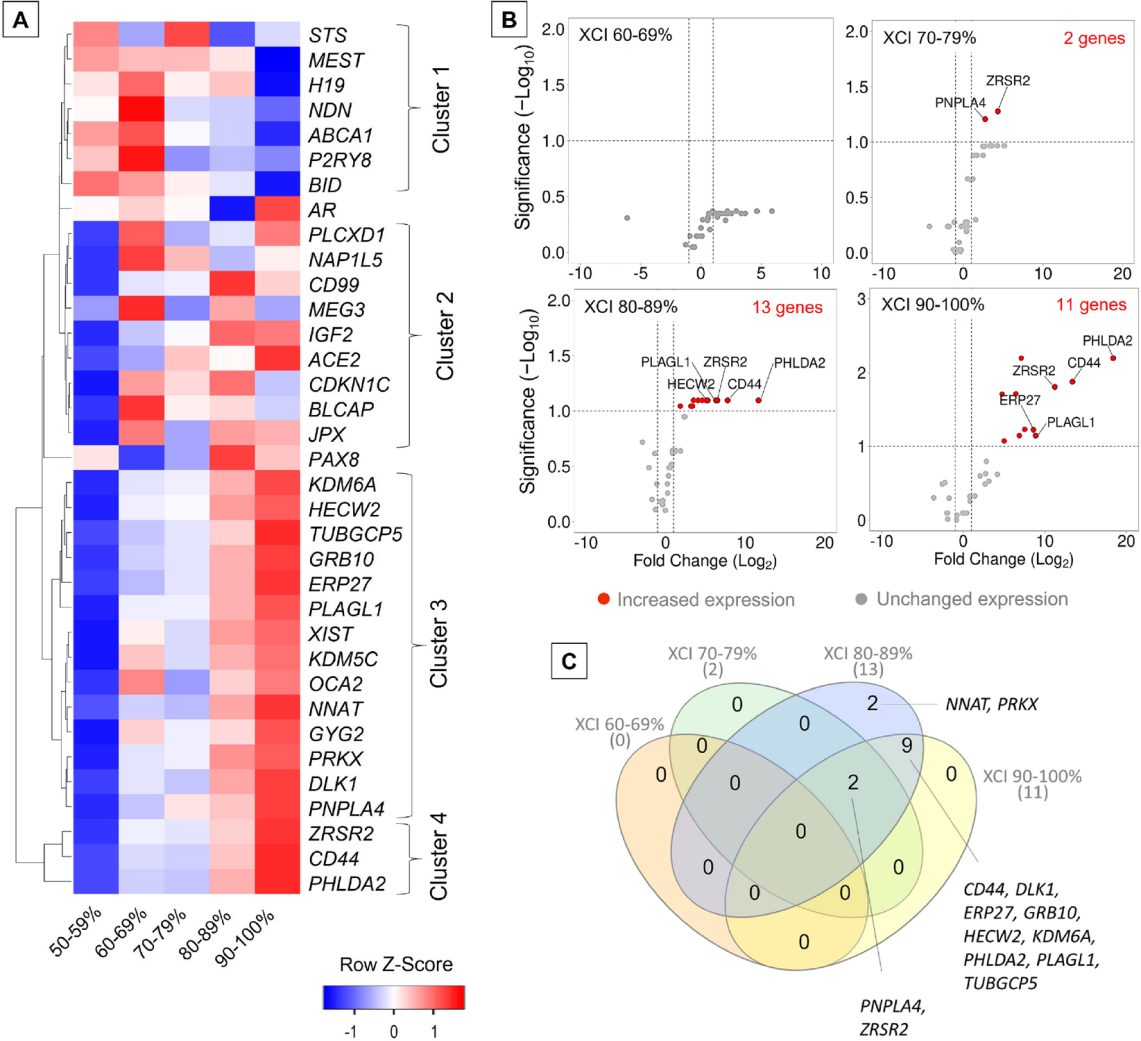
Gene expression profile of placental tissue of different X-chromosome inactivation (XCI) groups. **A** Heat map showing gene expression fold change in placental tissue; data are presented as row z-score. Hierarchical clustering is based on log_2_-transformed fold change values with the average linkage as the clustering method and the Euclidean distance measurement method. **B** Volcano plots present genes with significant differential expression in samples from groups with various XCI statuses. For each gene, the fold change (log_2_-transformed) was calculated against the expression determined for samples of the group with an XCI status of 50–59% (comparison group). Gene expression fold change threshold is set to log_2_FC =|1.0|, and the significance threshold is set to − log_10_(adjusted *P* value) = 1.0. The total number of genes with significantly increased expression is given in red in each of the graphs. **C** Venn diagram showing the distribution of shared upregulated genes in placental tissue in groups with different XCI skewing

Four major gene clusters with differential expression between the analysed XCI groups were observed for the placental tissue (heatmap in Fig. 4A). Cluster 1 consists of 7 genes that show general downregulation of gene expression with increasing XCI skewing, while clusters 3 and 4 show general upregulation of gene expression with increasing XCI skewing. Cluster 3 is the largest and consists of 14 genes, but it contains two distinct subclusters with disparate gene expression within the XCI 60–69% group (the expression of *XIST*, *KDM5C*, *OCA2*, and *GYG2* was upregulated in this group, while the other 10 genes were downregulated). Cluster 4, in terms of gene expression, is the most distant from the others.

For the samples of umbilical cord blood, three XCI skewing groups were identified: 50–59%, 60–69%, and 70–79%, and the last one consisted of only one sample with the highest detected XCI ratio of 73:27 (individual 507). Three major gene clusters of differential expression between these three XCI groups were observed (heatmap in Supplemental Fig. S1A) with an overall downregulation of gene expression in an individual from the 70–79% XCI group. The changes in gene expression observed between XCI 50–59% group and the other two groups were statistically insignificant (volcano plot in Supplemental Fig. S1B; Supplemental Table S2).

The log_2_-transformed fold change (log_2_FC) less than − 1.0 or higher than 1.0 was set as a threshold for downregulated or upregulated gene expression, respectively. In the placental tissue, gene expression did not change significantly between samples with XCI of 60–69% compared to samples with the most random XCI of 50–59%. Significant upregulation of gene expression was however detected in groups with XCI of 70–79%, 80–89%, and 90–100% (volcano plots in Fig. 4B, Supplemental Table S3)—transcript levels of two (*PNPLA4*, *ZRSR2*), 13 (*CD44*, *DLK1*, *ERP27*, *GRB10*, *HECW2*, *KDM6A*, *NNAT*, *PHLDA2*, *PLAGL1*, *PNPLA4*, *PRKX*, *TUBGCP5*, *ZRSR2*), and 11 genes (*CD44*, *DLK1*, *ERP27*, *GRB10*, *HECW2*, *KDM6A*, *PHLDA2*, *PLAGL1*, *PNPLA4*, *TUBGCP5*, *ZRSR2*), respectively, were increased. Downregulation of gene expression corresponding to increased XCI skewing was also observed, but none of it was significant. Among significantly upregulated genes, two were shared between placental samples from groups with XCI skewing of 70–79%, 80–89%, and 90–100%, while nine genes were shared only by the latter two groups; two genes were upregulated solely in XCI 80–89% group (Fig. 4C). The identified genes were annotated to biological process (BP) gene ontology (GO) terms such as signal transduction (GO:0,007,165; *GRB10*, *PRKX*, *DLK1*, *CD44*, *KDM6A*), cellular protein modification process (GO:0,006,464; *HECW2*, *NNAT*, *PRKX*, *CD44*, *KDM6A*), cell differentiation (GO:0,030,154; *PRKX*, *DLK1*, *KDM6A*), phosphorylation (GO:0,016,310; *GRB10*, *PRKX*, *CD44*), and cell migration (GO:0,016,477; *GRB10*, *PRKX*, *PHLDA2*, *CD44*).

**Fig. 5:**
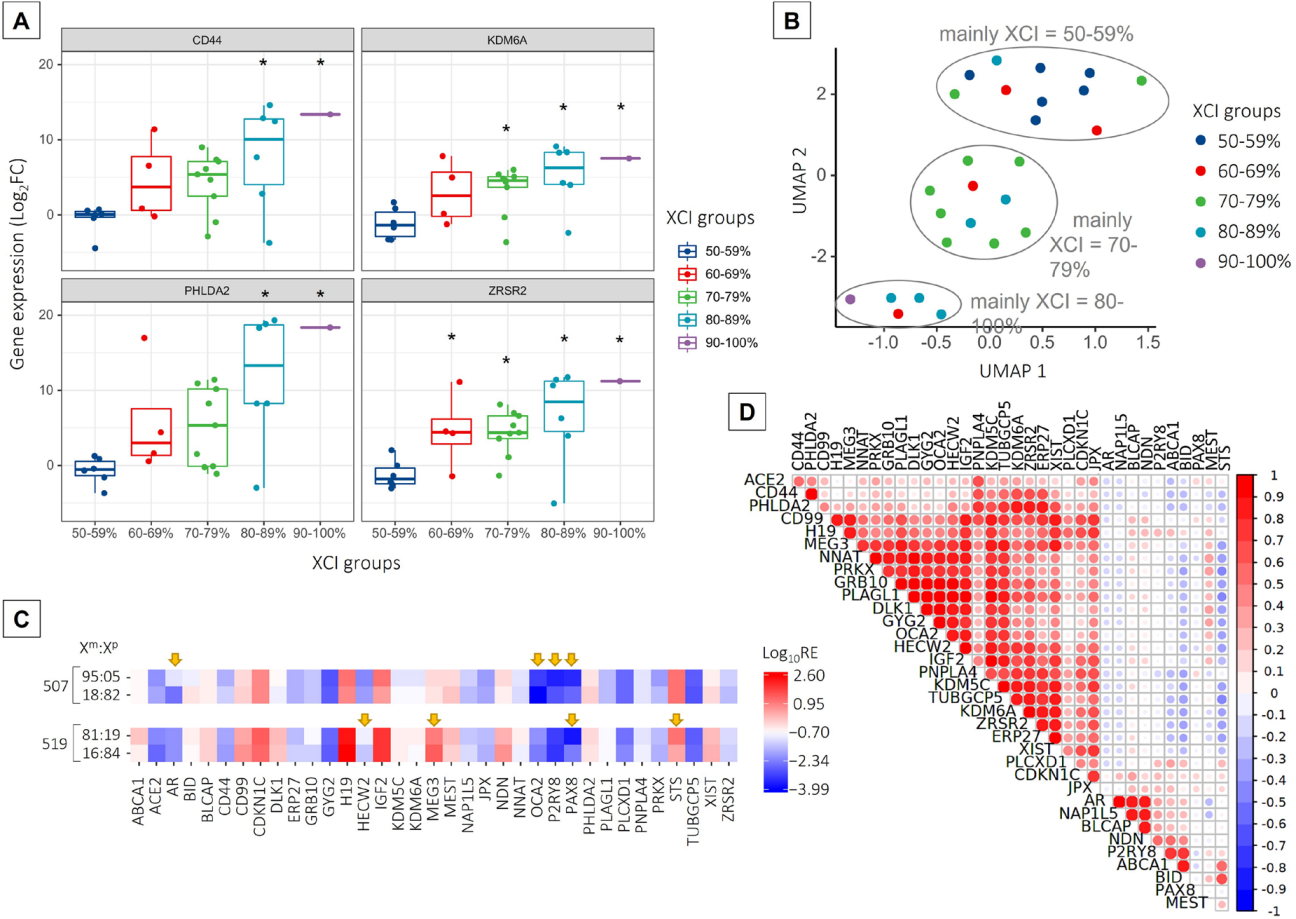
Changes in gene expression in placental tissue with increasing X-chromosome inactivation (XCI) skewing. **A** Box plots showing gene expression in placental tissue determined for XCI skewing groups. The box shows lower and upper quartiles and whiskers—minimum and maximum values of the data. The horizontal line inside the box represents median and dots—individual samples; the points located outside the whiskers represent outliers. One-way ANOVA with Fisher’s least significant difference post hoc analysis was performed. The black asterisk (*) denotes a significant (*P* value < 0.05) difference compared to the group with an XCI of 50–59%. **B** Uniform Manifold Approximation and Projection (UMAP) graph presenting the distribution of placental tissue samples from different XCI skewing groups according to the expression profiles of *CD44*, *KDM6A*, *PHLDA2*, and *ZRSR2* genes. **C** Gene expression profiles of two individuals (507 and 519) with the highest difference in the XCI ratios (shown on the left side of the heat maps) between placental tissue samples collected. The heat map shows log10-transformed, relative expression (RE) values obtained for each sample. Yellow arrows indicate differences in gene expression between both placental samples of an individual as identified by the Bland–Altman analysis of agreement between corresponding measurements (Bland–Altman plots are available in Supplemental Fig. S2). (D) Correlogram evidencing associations between gene expression in placental tissue. The correlation was assessed with parametric Pearson correlation analysis on relative expression (RE) data obtained for each gene. The size of the circles corresponds to Pearson’s *r* values. *R* values with corresponding *P* values are presented in Supplemental Table S4

In general, genes that were significantly upregulated in placental tissues from groups with XCI skewing > 80% showed very high fold change compared to the 50–59% XCI group; the log_2_FC values ranged from 1.84 up to 18.36 (Supplemental Table S3). One-way ANOVA showed that gene expression among all tested XCI groups differed significantly for *CD44*, *KDM6A*, *PHLDA2*, and *ZRSR2* (Fig. 5A). These genes showed very low expression in placental tissue samples characterized by random XCI of 50–59%; however, despite the presence of some outlier samples, they were significantly upregulated with an increasing degree of XCI skewing. Expression of *CD44* and *PHLDA2* was significantly upregulated in groups with XCI skewing of over 80%, while expression of *KDM6A *was significantly upregulated in groups with XCI skewing of over 70%. The *ZRSR2* gene expression increased significantly starting from the group with XCI skewing of 60% compared to the group with 50–59% XCI. Interestingly, the *CD44*, *PHLDA2*, and *ZRSR2* genes formed the most distant cluster 4 (compare the heatmap in Fig. 4A), while the *KDM6A* gene belonged to cluster 3 in which several other genes with significant differential expression were also clustered.

The Uniform Manifold Approximation and Projection (UMAP) analysis, which is a non-linear dimensionality reduction algorithm, was performed to visualize and cluster high-dimensional data. Using gene expression fold change obtained for *CD44*, *KDM6A*, *PHLDA2*, and *ZRSR2* genes as the input dataset, placental tissue samples were distributed into three distinct groups (Fig. 5B). One group tended to collocate samples with XCI of 50–59%; the second group, samples with XCI of 70–79%; and the third group, samples with XCI skewing in the range of 80–100%. There were some outliers, i.e., samples that belonged to a particular XCI skewing group; however, due to similarities in gene expression patterns, they collocated with samples belonging to other XCI skewing groups.

### Comparison of gene expression profile between placental tissue samples with the reversed pattern of X-chromosome inactivation

Two individuals (507 and 519) showed reversed skewed XCI ratios in two separate placental tissue samples—in one the paternally and, in the other, the maternally inherited X chromosome was preferentially inactivated (XCI of 18:82 and 95:05 for individual 507; XCI of 81:19 and 16:84 for individual 519). We aimed to find any differences in gene expression patterns between these samples by performing the analysis of agreement between relative gene expression values obtained for each of the genes profiled in the analysis performed on corresponding samples. For this purpose, we assumed both samples of an individual as replicates. For both individuals separately, the difference between the two relative expression measurements obtained for both samples was plotted against the average of these two measurements (Bland–Altman plot) (Supplemental Fig. S2); points scattered outside the estimated limits of agreement showed inconsistent expression levels in both examined samples of an individual. For both individuals, most of the analysed genes had similar levels of expression in corresponding samples despite the reversed XCI ratio (Supplemental Fig. S2; points plotted between the estimated limits of agreement). Gene expression levels were inconsistent between both samples only for several genes (Fig. 5C; genes denoted with yellow arrows); most of them were specific to an individual. However, for the *PAX8* gene, differences in expression levels between corresponding samples occurred in both individuals.

### Correlations between gene expression in placental tissue

Interestingly, significant correlations in gene expression for several genes profiled in placental tissue were also found (Fig. 5D, Supplemental Table S4). Relative gene expression determined by the RT-qPCR method most strongly correlated between *GYG2* and both *DLK1* and *GRB10*, *NAP1L5* and *AR*, *OCA2* and *DLK1*, *GRB10* and *GYG2*, and *PLAGL1* and both *GRB10* and *OCA2* genes (Pearson’s *r* > 0.95; *P* value < 0.05). Significant negative correlations were also identified between *PLAGL1* and *STS* genes, *PRKX*, and both *STS* and *TUBGCP5* genes; however, the correlations were only moderate (− 0.4 > Pearson’s *r* >  − 0.5; *P* value < 0.05).

## Discussion

In this study, we confirmed that XCI in healthy, naturally conceived females is not always a random process and skewing tends to change with females’ age. The XCI pattern varies between tissues, while the strongest correlation of the XCI ratios was established between saliva and blood. We also confirmed that the method of conception does not affect the XCI pattern. Finally, we have shown that the expression of certain genes involved in chromatin organization, cell proliferation, and growth is increased in the placental tissue with skewed XCI.

The random XCI pattern was the most common feature detected in females of all age groups and all examined tissues here; only 2% of the youngest (< 12 years) and 7% of the oldest (> 35 years) females showed the extremely skewed XCI of ≥ 90% in blood, buccal swabs, or saliva. These results are in agreement with previous studies, in which the XCI skewing ratio of ≥ 90:10 was identified in about 2–7% of females under 20–25 years old (Sharp et al. 2000; Amos-Landgraf et al. 2006; Kloska et al. 2011) and about 11–16% of female over 60 years (Sharp et al. 2000; Kloska et al. 2011). We also found a positive, albeit weak, correlation between the degree of XCI skewing and the age of females. The reported age at which the XCI ratio shifts toward more skewed differs between studies as it was shown to shift between 55 and 100 years (Kristiansen et al. 2005; Knudsen et al. 2007; Bolduc et al. 2008) or as soon as between 5 and 10 years of age (Wong et al. 2011). Although the increasing frequency of skewed XCI in females with age is suggested to be a natural consequence of ageing (Sandovici et al. 2004), the exact mechanism remains unclear.

Our findings also confirmed that the XCI skewing ratio in an individual varies between different tissues. Blood, buccal, and urinary epithelia were shown previously to have a similar degree of XCI in females under 25 years; however, in females over 60 years of age, the extremely skewed XCI of ≥ 90:10 was much more common in blood than in other tissues tested (Sharp et al. 2000). Here, the frequency of various XCI ratios in blood and buccal swabs from females of the three age groups was similar. Only in saliva, the XCI skewing of 80–90% and 90–100% was more frequent in females over 35 years.

We also found that the saliva XCI pattern correlates more strongly with the XCI in blood than the buccal swab. The composition of saliva is not constant, and one might assume that oral epithelial cells are most abundant. Meanwhile, 74% of the DNA isolated from saliva has been shown to originate from leukocytes (Thiede et al. 2000). Our analysis of the XCI pattern in blood, buccal epithelia, and saliva somewhat provides evidence to support the tissue composition of saliva.

Only a few females in our study had skewed XCI of 80–100% in more than one cell type simultaneously. Interestingly, some females showed XCI skewing in opposite directions in different tissues (including separate areas of the placenta); i.e. maternal X was favoured for inactivation in one tissue and paternal X in another tissue of the individual. In most cases, it was the buccal swab XCI ratio that was reversed compared to the XCI ratio in blood and saliva. To the best of our knowledge, this has not been reported previously; to date, only shifts in the XCI degree toward greater skewness have been noted in different tissues of a given individual (Sharp et al. 2000; Santos-Rebouças et al. 2020). The greatest difference in the XCI pattern was found in the oldest females, suggesting that the concordance of XCI statuses can weaken with age (Bittel et al. 2008).

Analysing the parent-of-origin effects on XCI skewing trait inheritance, we found that the XCI skewing in mothers does not correlate with that of the daughters; this seems to be in concordance with our previous observation that the X-inactivation locus (XIC) does not cosegregate with the XCI skewing trait suggesting an unknown but autosomally inherited factor to be linked to skewing (Kloska et al. 2011). This is particularly interesting regarding our results of upregulated expression of some autosomal genes such as *CD44* and *PHLDA2* associated with increased XCI skewing.

One clinically relevant question in our study concerned the effect of assisted reproductive technologies on the XCI outcome. The XCI occurs early in development, but its exact timing in humans is still inconclusive. According to some studies, the XCI process occurs in the morula and blastocyst stages before the implantation (van den Berg et al. 2009), while others postulate that XCI occurs after the blastocyst stage (Okamoto et al. 2011). Some studies report that in most female cells at embryonic day 7, X-linked genes are biallelically detected, and *XIST* RNA is expressed from and clouds both Xs (Petropoulos el.al. 2016; Reinius and Sandberg 2019), while others indicate a decrease in biallelic and increase in monoallelic expression of X-linked genes from the 4-cell to the blastocyst stage indicating that XCI is initiated during preimplantation development (Moreira de Mello et al. 2017). One might therefore be concerned whether in vitro culturing that is carried out, while using assisted reproductive technologies affects the XCI process. Our study provides evidence of no significant differences in the frequency of XCI skewing between age-matched, naturally, and IVF-conceived girls in either saliva, blood, or buccal swabs. In both groups, extreme XCI skewing of ≥ 90% was a rare feature found in less than 3% of cases per group. This is consistent with previous findings that in IVF- and naturally conceived females the frequency of extreme skewing of ≥ 90% (9.1% vs. 0, respectively) and skewing of 80–90% (9.1% vs. 6.5%, respectively) is not significantly different (Robinson et al. 2005; King et al. 2010).

Our findings confirm that the IVF techniques are unlikely to interfere with the XCI process. It is important to emphasize that our study is strengthened by examining XCI in different tissues of age-matched girls after IVF and those naturally conceived, whereas previous studies comparing these variables have only been performed for cord blood samples. We also recruited only young girls below 6 years to exclude the influence of the age-related increase of XCI skewing reported elsewhere (Wong et al. 2011). However, either here or in other studies, the sample size was relatively small (37 naturally- and 38 IVF-conceived girls in this study; between 22 and 74 individuals per group elsewhere); thus, caution to these findings must be applied, and further studies on larger cohorts are needed to verify the observations.

A major problem in XCI research is the definition of the skewing threshold set to classify XCI as skewed or extremely skewed. The threshold value is debatable and varies between studies which significantly affects the interpretation of results. For example, when the threshold is defined as XCI ≥ 70:30, 25–27% of the healthy females in a population are characterized by skewed XCI (Amos-Landgraf et al. 2006; Shvetsova et al. 2019), but when it is set to ≥ 65:35, nearly half of the females demonstrate this feature (Shvetsova et al. 2019). In our analyses, we did not adopt such a classification but instead used XCI intervals of 10% for grouping purposes. With this approach, we were able to analyse the gene expression patterns of the samples with the most convergent XCI status and compare them with the results obtained for the samples with the most random XCI status, thus avoiding discrepancies due to an arbitrary threshold of skewing. Using this approach, we were able to differentiate the XCI skewing groups according to the transcriptomic profiles, and we found that the expression of four genes—*CD44*, *KDM6A*, *PHLDA2*, and *ZRSR2*—is higher in samples with XCI ≥ 80%. Based on the expression of these four genes alone, the samples cluster into three groups—one including samples with XCI of 50–59%, the second including samples with XCI of 70–79%, and the third including samples with XCI ≥ 80%. Based on the transcriptomic profile determined in this study in placental tissue samples with different XCI ratios, we propose that the threshold considered as non-random be set to the XCI ≥ 80:20 as examination for transcription showed that only samples with XCI ≥ 80% differ significantly from those with the random XCI of 50–59%. Transcriptomic profiles of samples with XCI skewing of 60–69% and 70–79% are more similar to each other than to samples with XCI ≥ 80%.

To better characterize how the XCI process is regulated in human embryonic development, we examined the XCI pattern and gene expression in the placenta as a representative of extraembryonic tissue. The XCI was already proven to be random in the human placenta (Moreira de Mello et al. 2010), and the placenta was shown to consist of large patches of cells with an inactive X chromosome of either paternal or maternal origin (Moreira de Mello et al. 2010; Phung et al. 2022). Here, we confirmed that the XCI pattern varies greatly between different areas sampled from the foetal side of the same placenta. Regardless of the type of conception, patches with either random, skewed, or extremely skewed XCI ratios were identified in placentas. Interestingly, some placentas differed in the degree of XCI skewing between patches but not in the parental origin of the X selected for inactivation, while others showed a reversed XCI pattern with preferential inactivation of either paternal or maternal X depending on the patch. One unanticipated finding was that the expression level of the paired-box 8 (*PAX8*) gene was a common feature that differentiated placenta samples with reversed XCI in two individuals with this phenomenon. Transcription factor PAX8 is critical in the formation of tissues and organs during embryonic development (Khizer et al. 2021), but it also promotes the proliferation and survival of tumour cells when its expression is increased (Di Palma et al. 2013; Bie et al. 2019). Here, we hypothesize that changes in *PAX8* expression may affect the cell cycle or cell viability which in turn may contribute (as a secondary phenomenon) to skewing the XCI pattern after a primary XCI event.

Finally, our study provided evidence of differential expression of several genes involved in signal transduction, protein modification, phosphorylation, cell differentiation, and cell migration in association with increased XCI skewing in placental tissue. Notably, we found significantly increased expression of *CD44*, *KDM6A*, *PHLDA2*, and *ZRSR2* in samples with XCI skewing of over 80%. To our knowledge, these genes were not associated with the XCI skewing phenomenon to date. The *KDM6A* gene, encoding the lysine demethylase 6A (a histone demethylase) which mediates the removal of repressive methylation of histone H3K27 to establish transcriptionally permissive chromatin and takes a role in chromatin organization is especially interesting regarding the XCI. *KDM6A* escapes X inactivation and is a dosage-sensitive gene (Fagerberg et al. 2014; Chi et al. 2021). It has been shown that the mice *Kdm6a* (known as *Utx*) expression is lower from the inactive than active X chromosome (Xu et al. 2008). Here, with increasing XCI skewing, we observed a gradually increasing expression of *KDM6A*, and we hypothesize that this may interfere with the XCI process by mediating the demethylation of a particular X that, for some unknown reason, is more susceptible and consequently reducing the local efficiency of inactivation. If such expression was also characteristic at the beginning of an XCI event, it could be a factor modulating the efficiency of inactivation towards a particular X of the pair.

The products of *CD44*, *PHLDA2*, and *ZRSR2* genes are generally involved in cell growth, proliferation or migration. The CD44 molecule (Indian blood group), a cell-surface glycoprotein involved in cell–cell interactions, cell adhesion, and migration, plays a role in maintaining the placental structural integrity (Lambertini et al. 2012; Moore et al. 2015), while PHLDA2—the pleckstrin homology-like domain family A member 2—regulates the placenta growth (Tunster et al. 2010; Lambertini et al. 2012; Moore et al. 2015). Further, the loss of ZRSR2 (zinc finger CCCH-type, RNA-binding motif, and serine-/arginine-rich 2 protein), a component of the minor spliceosome (Garsetti et al. 2022), inhibits cell growth and alters the in vitro differentiation potential of haematopoietic cells (Madan et al. 2015). We hypothesize that certain levels of transcription of these genes may affect survival or growth rate resulting in the proliferative advantage of one pool of cells over the others in a mosaic tissue, ultimately leading to a substantial XCI skewing.

When analysing X-linked genes, it is also important to remember that some of them are subject to XCI while others escape XCI. The degree to which a gene escapes XCI varies for genes, tissues, developmental stages, and individuals. As suggested by Shvetsova et al. (2019), the presence of individuals with extreme XCI skewing should allow for analysis of the XCI escape of a gene, as, theoretically, an escapee gene should show a more balanced expression from both Xs than a gene subjected to XCI. Here, only some of the X-linked genes reported elsewhere as XCI escapees in the placenta (Phung et al. 2022) showed higher expression in placentas with XCI skewing of 80–100%, while expression of others was similar to that of the most random samples (XCI of 50–59%) (Supplemental Fig. S3). As two upregulated escapee genes, both coding for histone demethylases (KDM5C and KDM6A), are involved in transcription regulation and chromatin remodelling, any changes in their transcription may be responsible for the preferential inactivation of the X-chromosome carrying an allele characterized by higher expression (or vice versa). Similarly, only for some subject-to-XCI genes, we have found the differential expression in tissues with random vs. skewed XCI (Supplemental Fig. S3). It seems therefore that skewing does not uniformly affect the expression of either all subject-to-XCI genes or all XCI-escapee genes suggesting other mechanisms involved in this variation.

We are aware of limitations to our gene expression study, as it was performed on full-term placenta, while the XCI process occurs in early development. Thus, the gene expression patterns established here may be different from those that occur around the time of the XCI event. Furthermore, we determined expression profiles for a small number of placenta samples, and our approach was limited to examining only transcripts and not protein abundance. However, the differences in gene expression patterns between samples with random and skewed XCI provide new insights into the factors contributing to deviations from XCI randomness and pinpoint new directions for future studies of the XCI process.

In conclusion, our study confirmed that X-chromosome inactivation patterns in humans depend on age and tissue but not on the method of conception. In our opinion, the placenta, due to its patchy X inactivation, appears to be a promising tissue type for transcriptomic and proteomic studies to deepen our understanding of the X-chromosome inactivation skewing phenomenon.

## Supplementary Information

Below is the link to the electronic supplementary material.

Supplementary file1 (DOCX 383 KB)

Supplementary file2 (XLSX 30 KB)

Supplementary file3 (DOCX 657 KB)

## Data Availability

Data and materials are available on request.

## Abbreviations

IVF: In vitro fertilization
XCI: X-chromosome inactivation

## References

[CR1] Allen RC, Zoghbi HY, Moseley a B, et al (1992) Methylation of HpaII and HhaI sites near the polymorphic CAG repeat in the human androgen-receptor gene correlates with X chromosome inactivation. Am J Hum Genet 51:1229–39 PMC1682906 1281384

[CR2] Amos-Landgraf JM, Cottle A, Plenge RM (2006). X chromosome–inactivation patterns of 1,005 phenotypically unaffected females. Am J Human Genet.

[CR3] Au W-Y, Ma ESK, Lam VMS (2004). Glucose 6-phosphate dehydrogenase (G6PD) deficiency in elderly Chinese women heterozygous for G6PD variants. Am J Med Genet.

[CR4] Ayllon-Benitez A, Bourqui R, Thébault P, Mougin F (2020) GSAn: an alternative to enrichment analysis for annotating gene sets. NAR Genom Bioinform 2:lqaa017. 10.1093/nargab/lqaa017 10.1093/nargab/lqaa017 PMC7671311 33575577

[CR5] Babicki S, Arndt D, Marcu A (2016). Heatmapper: web-enabled heat mapping for all. Nucleic Acids Res.

[CR6] Beever C, Lai BPY, Baldry SEL (2003). Methylation of ZNF261 as an assay for determining X chromosome inactivation patterns. Am J Med Genet A.

[CR7] Bie L-Y, Li D, Wei Y (2019). SOX13 dependent PAX8 expression promotes the proliferation of gastric carcinoma cells. Artif Cells Nanomed Biotechnol.

[CR8] Bittel DC, Theodoro MF, Kibiryeva N (2008). Comparison of X-chromosome inactivation patterns in multiple tissues from human females. J Med Genet.

[CR9] Bolduc V, Chagnon P, Provost S (2008). No evidence that skewing of X chromosome inactivation patterns is transmitted to offspring in humans. J Clin Investig.

[CR10] Cazzola M, May A, Bergamaschi G (2000). Familial-skewed X-chromosome inactivation as a predisposing factor for late-onset X-linked sideroblastic anemia in carrier females. Blood.

[CR11] Chi Y-I, Stodola TJ, De Assuncao TM (2021). Molecular mechanics and dynamic simulations of well-known Kabuki syndrome-associated KDM6A variants reveal putative mechanisms of dysfunction. Orphanet J Rare Dis.

[CR12] Danger R, Moiteaux Q, Feseha Y, et al (2021) FaDA: a web application for regular laboratory data analyses. PLoS One 16:e0261083. 10.1371/journal.pone.0261083 10.1371/journal.pone.0261083 PMC8687579 34928943

[CR13] de Hoon B, Monkhorst K, Riegman P (2015). Buccal swab as a reliable predictor for X inactivation ratio in inaccessible tissues. J Med Genet.

[CR14] Di Palma T, Filippone MG, Pierantoni GM (2013). Pax8 has a critical role in epithelial cell survival and proliferation. Cell Death Dis.

[CR15] Fagerberg L, Hallström BM, Oksvold P (2014). Analysis of the human tissue-specific expression by genome-wide integration of transcriptomics and antibody-based proteomics. Mol Cell Proteomics.

[CR16] Garsetti DE, Sahay K, Wang Y, Rogers MB (2022). Sex and the basal mRNA synthesis machinery. Wires RNA.

[CR17] Goedhart J, Luijsterburg MS (2020). VolcaNoseR is a web app for creating, exploring, labeling and sharing volcano plots. Sci Rep.

[CR18] Goedhart J, Rishniw M (2021). BA-plotteR – a web tool for generating Bland-Altman plots and constructing limits of agreement. Res Vet Sci.

[CR19] Goto T, Monk M (1998). Regulation of X-chromosome inactivation in development in mice and humans. Microbiol Mol Biol Rev.

[CR20] Heberle H, Meirelles GV, da Silva FR (2015). InteractiVenn: a web-based tool for the analysis of sets through Venn diagrams. BMC Bioinformatics.

[CR21] Juchniewicz P, Kloska A, Tylki-Szymańska A (2018). Female Fabry disease patients and X-chromosome inactivation. Gene.

[CR22] Juchniewicz P, Piotrowska E, Kloska A (2021). Dosage compensation in females with X-linked metabolic disorders. Int J Mol Sci.

[CR23] Kappil MA, Green BB, Armstrong DA (2015). Placental expression profile of imprinted genes impacts birth weight. Epigenetics.

[CR24] Katari S, Turan N, Bibikova M (2009). DNA methylation and gene expression differences in children conceived in vitro or in vivo. Hum Mol Genet.

[CR25] Khizer K, Padda J, Khedr A (2021). Paired-box gene 8 (PAX8) and its association with epithelial carcinomas. Cureus.

[CR26] King JL, Yang B, Sparks AE, Mains LM, Murray JC, Van Voorhis BJ (2010) Skewed X inactivation and IVF-conceived infants. Reprod Biomed Online 20(5):660–663. 10.1016/j.rbmo.2010.01.011 10.1016/j.rbmo.2010.01.011 PMC2923043 20207584

[CR27] Kleijkers SHM, Eijssen LMT, Coonen E (2015). Differences in gene expression profiles between human preimplantation embryos cultured in two different IVF culture media. Hum Reprod.

[CR28] Kloska A, Jakóbkiewicz-Banecka J, Tylki-Szymańska A (2011). Female Hunter syndrome caused by a single mutation and familial XCI skewing: implications for other X-linked disorders. Clin Genet.

[CR29] Knoppert D, Reed M, Benavides S, et al (2007) Paediatric age categories to be used in differentiating between listing on a model essential medicines list for children. Position Paper 1–5

[CR30] Knudsen GPS, Pedersen J, Klingenberg O (2007). Increased skewing of X chromosome inactivation with age in both blood and buccal cells. Cytogenet Genome Res.

[CR31] Kristiansen M, Knudsen GPS, Bathum L (2005). Twin study of genetic and aging effects on X chromosome inactivation. Eur J Hum Genet.

[CR32] Lambertini L, Marsit CJ, Sharma P (2012). Imprinted gene expression in fetal growth and development. Placenta.

[CR33] Litzky JF, Deyssenroth MA, Everson TM (2017). Placental imprinting variation associated with assisted reproductive technologies and subfertility. Epigenetics.

[CR34] Livak KJ, Schmittgen TD (2001). Analysis of relative gene expression data using real-time quantitative PCR and the 2(-Delta Delta C(T)) Method. Methods.

[CR35] Lyon MF (1961). Gene action in the X-chromosome of the mouse (mus musculus L.). Nature.

[CR36] Madan V, Kanojia D, Li J (2015). Aberrant splicing of U12-type introns is the hallmark of ZRSR2 mutant myelodysplastic syndrome. Nat Commun.

[CR37] Market-Velker BA, Zhang L, Magri LS (2010). Dual effects of superovulation: loss of maternal and paternal imprinted methylation in a dose-dependent manner. Hum Mol Genet.

[CR38] Migeon BR (2020). X-linked diseases: susceptible females. Genet Med.

[CR39] Moore GE, Ishida M, Demetriou C (2015). The role and interaction of imprinted genes in human fetal growth. Philosophical Transactions of the Royal Society b: Biological Sciences.

[CR40] Moreira de Mello JC, Fernandes GR, Vibranovski MD, Pereira LV (2017). Early X chromosome inactivation during human preimplantation development revealed by single-cell RNA-sequencing. Sci Rep.

[CR41] Moreira de Mello JC, de Araújo ÉSS, Stabellini R, et al (2010) Random X inactivation and extensive mosaicism in human placenta revealed by analysis of allele-specific gene expression along the X chromosome. PLoS One 5:e10947. 10.1371/journal.pone.0010947 10.1371/journal.pone.0010947 PMC2881032 20532033

[CR42] Nelissen ECM, Dumoulin JCM, Busato F (2014). Altered gene expression in human placentas after IVF/ICSI. Hum Reprod.

[CR43] Okamoto I, Patrat C, Thépot D (2011). Eutherian mammals use diverse strategies to initiate X-chromosome inactivation during development. Nature.

[CR44] Ørstavik KH (2009) X chromosome inactivation in clinical practice. Hum Genet 363–373 10.1007/s00439-009-0670-5 19396465

[CR45] Patrat C, Ouimette J-F, Rougeulle C (2020) X chromosome inactivation in human development. Development 147:dev183095. 10.1242/dev.183095 10.1242/dev.183095 31900287

[CR46] Petropoulos S, Edsgärd D, Reinius B (2016). Single-cell RNA-seq reveals lineage and X chromosome dynamics in human preimplantation embryos. Cell.

[CR47] Pfaffl MW, Tichopad A, Prgomet C, Neuvians TP (2004). Determination of stable housekeeping genes, differentially regulated target genes and sample integrity: BestKeeper–Excel-based tool using pair-wise correlations. Biotechnol Lett.

[CR48] Phung TN, Olney KC, Pinto BJ, et al (2022) X chromosome inactivation in the human placenta is patchy and distinct from adult tissues. Human Genetics and Genomics Advances 3:100121. 10.1016/j.xhgg.2022.100121 10.1016/j.xhgg.2022.100121 PMC9194956 35712697

[CR49] Plenge RM, Stevenson RA, Lubs HA (2002). Skewed X-chromosome inactivation is a common feature of X-linked mental retardation disorders. Am J Hum Genet.

[CR50] Reinius B, Sandberg R (2019) Expression reduction of biallelically transcribed X-linked genes during the human female preimplantation development. bioRxiv 682286

[CR51] Robinson WP, Peñaherrera MS, Gair J, Hatakeyama C, Ma S (2005) X-chromosome inactivation and telomere size in newborns resulting from intracytoplasmic sperm injection. Am J Med Genet A 137A(3):343–345. 10.1002/ajmg.a.30886 10.1002/ajmg.a.30886 16097000

[CR52] Sandovici I, Naumova AK, Leppert M (2004). A longitudinal study of X-inactivation ratio in human females. Hum Genet.

[CR53] Santos-Rebouças CB, Boy R, Vianna EQ, et al (2020) Skewed X-chromosome inactivation and compensatory upregulation of escape genes precludes major clinical symptoms in a female with a large Xq deletion. Front Genet 11:. 10.3389/fgene.2020.00101 10.3389/fgene.2020.00101 PMC7064548 32194616

[CR54] Sharp A, Robinson D, Jacobs P (2000). Age- and tissue-specific variation of X chromosome inactivation ratios in normal women. Hum Genet.

[CR55] Shvetsova E, Sofronova A, Monajemi R (2019). Skewed X-inactivation is common in the general female population. Eur J Hum Genet.

[CR56] Tan K, An L, Miao K (2016). Impaired imprinted X chromosome inactivation is responsible for the skewed sex ratio following in vitro fertilization. Proc Natl Acad Sci.

[CR57] Thiede C, Prange-Krex G, Freiberg-Richterm J (2000). Buccal swabs but not mouthwash samples can be used to obtain pretransplant DNA fingerprints from recipients of allogeneic bone marrow transplants. Bone Marrow Transplant.

[CR58] Tukiainen T, Villani A-C, Yen A (2017). Landscape of X chromosome inactivation across human tissues. Nature.

[CR59] Tunster SJ, Tycko B, John RM (2010). The Imprinted Phlda2 gene regulates extraembryonic energy stores. Mol Cell Biol.

[CR60] van den Berg IM, Laven JSE, Stevens M (2009). X chromosome inactivation is initiated in human preimplantation embryos. Am J Hum Genet.

[CR61] Wong CCY, Caspi A, Williams B, et al (2011) A longitudinal twin study of skewed X chromosome-inactivation. PLoS One 6:e17873. 10.1371/journal.pone.0017873 10.1371/journal.pone.0017873 PMC3062559 21445353

[CR62] Wu EX, Stanar P, Ma S (2014). X-chromosome inactivation in female newborns conceived by assisted reproductive technologies. Fertil Steril.

[CR63] Xu J, Deng X, Watkins R, Disteche CM (2008). Sex-specific differences in expression of histone demethylases Utx and Uty in mouse brain and neurons. J Neurosci.

