# Supplementary material for: X-chromosome inactivation patterns depend on age and tissue but not conception method in humans
Source: Chromosome Res. 2023 Jan 25;31(1):4. doi: 10.1007/s10577-023-09717-9 (PMC9877087; doi:10.1007/s10577-023-09717-9)
Supplement: Supplementary file 1 — Supplementary file1 (DOCX 383 KB) [file 10577_2023_9717_MOESM1_ESM.docx]

**X chromosome inactivation patterns depend on age and tissue but not conception method in humans**

Patrycja Juchniewicz^1^, Anna Kloska^1*^, Karolina Portalska^2^, Joanna Jakóbkiewicz-Banecka^1^, Grzegorz Węgrzyn^2^, Joanna Liss^3,1^, Piotr Głodek^3^, Stefan Tukaj^2^, Ewa Piotrowska^2*^

^1^ Department of Medical Biology and Genetics, Faculty of Biology, University of Gdańsk, Gdańsk, Poland

^2^ Department of Molecular Biology, Faculty of Biology, University of Gdańsk, Gdańsk, Poland

^3^ Research and Development Center, Sopot, Poland

^*^**Correspondence:**

Ewa Piotrowska, University of Gdańsk, Department of Molecular Biology, Wita Stwosza 59, 80-308 Gdańsk, Poland. Tel. +48 58 523 6040; e-mail: ewa.piotrowska@ug.edu.pl

Anna Kloska, University of Gdańsk, Department of Medical Biology and Genetics, Wita Stwosza 59, 80-308 Gdańsk, Poland. Tel. +48 58 523 6044; e-mail: anna.kloska@ug.edu.pl

**Supplemental Tables**

**Supplemental Table S1.** Characteristics of genes analysed in this study for expression in placental tissue samples.

| **Gene** | **Full name** | **OMIM Gene ID** | **Imprinting/XCI status in placenta** | **Function** | **Expression in foetal tissue and placenta** | **Reference** |
| --- | --- | --- | --- | --- | --- | --- |
| *ABCA1* | ATP Binding Cassette Subfamily A Member 1 | 600046 | Belongs to the group of imprinted genes regulating fetoplacental development/subject to XCI | Catalyses the translocation of phospholipids from the cytoplasmic to the exocytoplasmic leaflet of membranes coupled to the hydrolysis of ATP; participates in phospholipid transfer to apolipoproteins to form nascent high-density lipoproteins/HDLs | High expression in the human placenta; different placental expressed by birth category: upregulated in large for gestational age infants compared to small for gestational age infants | (Baumann et al. 2013; Kappil et al. 2015; Phung et al. 2022) |
| *ACE2* | Angiotensin-Converting Enzyme 2 | 300335 | ND/subject to XCI | Catalyses the cleavage of angiotensin I into angiotensin 1-9, and angiotensin II into the vasodilator angiotensin 1-7; a functional receptor for the spike glycoprotein of the human coronavirus HCoV-NL63 and the human severe acute respiratory syndrome coronaviruses, SARS-CoV and SARS-CoV-2 | Expressed in a membranous pattern in the cytotrophoblast (CT), extravillous trophoblast (EVT), and in the syncytiotrophoblast (ST) of the chorionic villi with expression highest on the stromal side of the ST. | (Hecht et al. 2020; Phung et al. 2022) |
| *AR* | Androgen Receptor | 313700 | ND/subject to XCI | Nuclear receptor that functions as a steroid-hormone activated transcription factor; activated by binding any of the androgenic hormones, including testosterone and dihydrotestosterone in the cytoplasm and then translocating into the nucleus | Expressed in male reproductive organs, such as efferent ductules, urogenital sinus, Wolffian ducts (WDs), epididymides, ductus deferens, seminal vesicles (SVs), coagulating glands, prostates, and bulbourethral glands and expressed in various ovarian cell types; expressed in placentas in near-term and term pregnancy | (Walters et al. 2012; Cotton et al. 2013; Davey and Grossmann 2016; Phung et al. 2022) |
| *BID* | BH3 Interacting Domain Death Agonist | 601997 | ND/NA (chr 22) | Pro-apoptotic member of the BCL-2 family of cell death regulators that regulate the permeabilization of the outer mitochondrial membrane; mediator of mitochondrial damage induced by caspase-8 (CASP8) | Dynamic expression in embryonic and postnatal brain, indicating the important role in neural development; expressed of isoform 2 in spleen, pancreas, and placenta | (Krajewska et al. 2002; Renshaw et al. 2004) |
| *BLCAP* | BLCAP Apoptosis Inducing Factor | 613110 | Imprinted in brain where different transcript variants are expressed from each parental allele. Transcript variants initiating from the upstream promoter are expressed preferentially from the maternal allele, while transcript variants initiating downstream of the interspersed NNAT gene are expressed from the paternal allele/NA (chr 20) | Reduces cell growth by stimulating apoptosis; regulator of cell proliferation and coordinate apoptosis and cell cycle progression via a novel mechanism independent of both p53/TP53 and NF-kappa-B. | Imprinted in fetal brain; relatively low level of expression in cervix, bladder, testis, placenta | (Hamed et al. 2012) |
| *CD44* | CD44 Molecule (Indian Blood Group) | 107269 | Imprinted gene in fetoplacental development indicated by the Ingenuity Pathway Analysis (IPA)/NA (chr 11) | Cell-surface glycoprotein involved in cell-cell interactions, cell adhesion and migration; receptor for hyaluronic acid (HA); mediates cell-cell and cell-matrix interactions through its affinity for HA; interact with other ligands, such as osteopontin, collagens, and matrix metalloproteinases (MMPs); participates in a wide variety of cellular functions including lymphocyte activation, recirculation and homing, haematopoiesis, and tumour metastasis. | Role in the stabilization and orientation of the HA network in the human placenta, important in maintaining the placental structural integrity | (Lambertini et al. 2012; Moore et al. 2015) |
| *CD99* | CD99 Molecule (Xg Blood Group) | 313470 | ND/XCI escapee (variable) | Involved in T-cell adhesion processes, spontaneous rosette formation with erythrocytes, leukocyte migration, T-cell adhesion, ganglioside GM1 and transmembrane protein transport | upregulation in cytotrophoblasts from  across gestation in male fetuses compared to females | (Phung et al. 2022; Braun et al. 2022) |
| *CDKN1C* | Cyclin Dependent Kinase Inhibitor 1C | 600856 | Imprinted, with preferential expression of the maternal allele /NA (chr 11) | Strong inhibitor of several G1 cyclin/Cdk complexes and a negative regulator of cell proliferation | Negative regulator of foetal growth; monoallelically expressed in both placenta and fetal tissues | (Yuen et al. 2016; Berland et al. 2022) |
| *DLK1* | Delta Like Non-Canonical Notch Ligand 1 | 176290 | Imprinted gene in fetoplacental development highly expressed in the placenta, predominantly expressed from the paternally inherited chromosome during foetal development/NA (chr 14) | Epidermal growth factor; transmembrane protein functions as a regulator of cell growth; involved in the differentiation of several cell types including adipocytes | Highly expressed by the placenta, specifically in stromal cells of the placental villi that are in close contact with the vasculature, expressed by foetal tissues and endocrine tissues; required for maternal metabolic adaptations to pregnancy; associated with foetal growth restriction | (Lambertini et al. 2012; Moore et al. 2015; Cleaton et al. 2016) |
| *ERP27* | Endoplasmic Reticulum Protein, 27-KD | 610642 | ND/NA (chr 12) | Noncatalytic member of the protein disulphide isomerase (PDI) family of endoplasmic reticulum (ER) proteins; specifically binds unfolded proteins and may recruit protein disulphide isomerase PDIA3 to unfolded substrates | Expressed in a variety of tissue types, including in the spleen, lung, kidney, thymus, and bone marrow and most highly in the pancreas; cDNA sources include cervix, breast, placenta, colon, lung, pancreas, thyroid, and bone marrow | (Alanen et al. 2006) |
| *GRB10* | Growth Factor Receptor Bound Protein 10 | 601523 | Imprinted in an isoform- and tissue-specific manner, with expression observed from the paternal allele in the brain, and the maternal allele in the placental trophoblasts/NA (chr 7) | Growth factor receptor-binding protein that interacts with insulin receptors and insulin-like growth-factor receptors; implicated in the regulation of cell proliferation, apoptosis, and metabolism | Highly expressed in the placenta; acts to limit the placental size and efficiency; expressed from the maternal allele in human placental villous trophoblasts; expression positively associated with placental weight and birth weight | (Moore et al. 2015; Plasschaert and Bartolomei 2015) |
| *GYG2* | Glycogenin 2 | 300198 | ND/subject to XCI | Self-glycosylating protein covalently linked to glycogen that acts as a primer for the action of glycogen synthase; regulatory role in glucose and lipid metabolism in adipose tissue and an extended role in whole-body glucose homeostasis | Strongly expressed in the human liver and moderately expressed in the brain, heart, pancreas, and kidney from foetal to adult stages | (Imagawa et al. 2014; Phung et al. 2022) |
| *H19* | H19 Imprinted Maternally Expressed Transcript | 103280 | Imprinted gene in fetoplacental development highly expressed in the placenta; expressed only from the maternally inherited chromosome/NA (chr 11) | Long non-coding RNA which functions as a tumour suppressor; controls the expression of several genes involved in the growth control of the embryo | Strongly expressed during embryogenesis and in endoderm and mesoderm derived tissues during foetal life | (Monnier et al. 2013; Moore et al. 2015) |
| *HECW2* | HECT, C2 and WW Domain Containing E3 Ubiquitin Protein Ligase 2 | 617245 | ND/NA (chr 2) | Member of a family of HECT-type ubiquitin ligases which plays an important role in the proliferation, migration, and differentiation of neural crest cells as a regulator of glial cell line-derived neurotrophic factor (GDNF)/Ret signalling and regulates the metaphase-to-anaphase transition during the cell cycle | Highly expressed in the placenta | (Fagerberg et al. 2014; Berko et al. 2017) |
| *IGF2* | Insulin Like Growth Factor 2 | 147470 | Imprinted gene, expressed only from the paternal allele/NA(chr 11) | Regulator of fetoplacental development; involved in tissue differentiation. and in glucose metabolism in adipose tissue, skeletal muscle | Highly expressed in the fetus and placenta; highly expressed by the villous and extravillous cytotrophoblasts during the first trimester and the extravillous cytotrophoblasts during the third trimester | (Harris et al. 2011; Aykroyd et al. 2020) |
| *JPX* | JPX Transcript, XIST Activator  (Just Proximal To XIST) | 300832 | Imprinted expression from the Xp chromosome/subject to XCI | Nonprotein-coding RNA transcribed from a gene within the X-inactivation centre (XIC) that appears to participate in X chromosome inactivation | Significantly upregulated in females; the earliest gene to be activated in preimplantation embryos, before XIST | (Patrat et al. 2020; Phung et al. 2022) |
| *KDM5C* | Lysine Demethylase 5C | 314690 | ND/XCI escapee | Specific H3K4me3 and H3K4me2 demethylase, and acts as a transcriptional repressor through the RE-1-silencing transcription factor (REST) complex | Female-upregulated X-linked gene; overexpressed in retina, placenta, heart, and adipocyte | (Gonzalez et al. 2018; Phung et al. 2022) |
| *KDM6A* | Lysine Demethylase 6A | 300128 | ND/XCI escapee | Mediates removal of repressive trimethylation and dimethylation but not monomethylation of histone H3 lys27 to establish transcriptionally permissive chromatin; regulation of posterior development, by regulating HOX gene expression | Female-upregulated X-linked genes; overexpressed in plasma, placenta, CD8 T cells; | (Fagerberg et al. 2014; Chi et al. 2021; Phung et al. 2022) |
| *MEG3* | Maternally Expressed 3 | 605636 | Imprinted gene in fetoplacental development highly expressed in the placenta, maternally expressed/NA (chr 14) | Encodes a non-coding RNA; acts as a growth suppressor in tumour cells; interacts with the tumour suppressor p53, and regulates p53 target gene expression; plays important roles in the regulation of diverse cellular processes, such as apoptosis, proliferation, and migration | Highly expressed in human placenta tissues; participate in regulating placental vascular development | (Zhang et al. 2003; Lambertini et al. 2012; Moore et al. 2015) |
| *MEST* | Mesoderm Specific Transcript | 601029 | Imprinted gene in fetoplacental development highly expressed in the placenta; preferential expression from the paternal allele in foetal tissues, and isoform-specific imprinting in lymphocytes/NA (chr 7) | Member of the alpha/beta hydrolase superfamily; plays an important role in regulating foetal growth and development and placental function; negative regulator of human adipocyte differentiation; involved in mammary gland maturation | Expressed in all foetal tissues, including the brain, skeletal muscle, kidney, adrenal, tongue, heart, skin, and placenta; regulates growth and functioning of the placenta | (Moore et al. 2015; Yonekura et al. 2019) |
| *NAP1L5* | Nucleosome Assembly Protein 1 Like 5 | 612203 | Tissue-specific imprinting with expression from the paternal allele/NA (chr 4) | Histone chaperone that plays an important role in gene transcription; required for the development of cardiomyocyte hypertrophy; regulation of nucleolus hypertrophy, ribosome assembly and protein synthesis; translation control during the pathological growth of cardiomyocytes | Paternally expressed in human fetal  spinal cord, brain, heart, and tongue | (Wood et al. 2007; Guo et al. 2021) |
| *NDN* | Necdin | 602117 | Maternally imprinted/NA (chr 15) | Growth suppressor that facilitates the entry of the cell into cell cycle arrest; binds to and represses the activity of cell-cycle-promoting proteins such as SV40 large T antigen, adenovirus E1A, and the transcription factor E2F; interacts with p53 to inhibit cell growth; functions as a transcription factor and binds directly to specific guanosine-rich DNA sequences | Linked to pregnancy complications; plays a role in neuronal differentiation at an early stage of development; downregulation of placental expression among preterm preeclampsia placentas | (Deyssenroth et al. 2020) |
| *NNAT* | Neuronatin | 603106 | Imprinted gene in fetoplacental development indicated by the Ingenuity Pathway Analysis (IPA); Preferentially imprinted maternally allele/NA (chr 20) | Proteolipid involved in the regulation of ion channels during brain development; forming and maintaining the structure of the nervous system; increased insulin secretion by regulating intracellular calcium levels and hyperglycemia-induced apoptosis in pancreatic β-cells | Imprinted in placenta and brain; increased expression in developing placentas | (Gu et al. 2012; Moore et al. 2015; Gao et al. 2016) |
| *OCA2* | OCA2 Melanosomal Transmembrane Protein | 611409 | Maternally imprinted in the placenta/NA (chr 15) | Integral membrane protein involved in small molecule transport, specifically tyrosine, which is a precursor to melanin synthesis; involved in mammalian pigmentation; regulates the pH of melanosome and the melanosome maturation | Transcripts found in primary melanocytes and foetal brain | (Hivert et al. 2020) |
| *P2RY8* | P2Y Receptor Family Member 8 | 300525 | ND/subject to XCI | Belongs to the P2Y family of G-protein coupled receptors that are preferentially activated by adenosine and uridine nucleotides; mediates the inhibition of migration and regulates the growth of B cells in lymphoid tissues | Transcripts found in the placenta | (Lu et al. 2019; Phung et al. 2022) |
| *PAX8* | Paired Box 8 | 167415 | ND/NA (chr 2) | Transcription factors involved in thyroid follicular cell development and expression of thyroid-specific genes | Expressed in the human placenta at term, mainly in the nuclei of the trophoblast cells | (Ferretti et al. 2005) |
| *PHLDA2* | Pleckstrin Homology-Like Domain Family A Member 2 | 602131 | Imprinted gene in fetoplacental development highly expressed in the placenta, maternally expressed/NA (chr 11) | Plays a role in regulating placenta growth | Predominantly expressed in the placenta from the maternal allele being expressed in syncytiotrophoblast layers II and III of the labyrinth | (Lambertini et al. 2012; Moore et al. 2015) |
| *PLAGL1* | PLAG1 Like Zinc Finger 1 | 603044 | Imprinted gene in fetoplacental development, paternally expressed/NA (chr 6) | Plays roles as transcription factor as well as a cofactor of other regulator proteins; tumour suppressor gene, whose product regulates apoptosis and cell cycle arrest | Highly expressed in the placenta; regulates overlapping gene networks in placental trophoblast and endothelial cells, and may play a critical role in placental development | (Moore et al. 2015; Vega-Benedetti et al. 2017; Starks et al. 2020) |
| *PLCXD1* | Phosphatidylinositol Specific Phospholipase C X Domain Containing 1 | 300974 | As PAR gene might be subject to genomic imprinting /XCI escapee | Receptor-regulated phosphodiesterases; regulator of cytosolic calcium and/or the activity of protein kinases | Highly expressed in the placenta | (Navarro-Cobos et al. 2020; Phung et al. 2022) |
| *PNPLA4* | Patatin Like Phospholipase Domain Containing 4 | 300102 | ND/subject to XCI | Triacylglycerol lipase and transacylase activities; involved in adipocyte triglyceride homeostasis | Overexpressed in foetal heart, foetal testis, ovary; mRNA expression in embryonic tissues: ovary, testis, blood | (Hermansson et al. 2016; Phung et al. 2022) |
| *PRKX* | Protein Kinase X-Linked | 300083 | ND/subject to XCI | Serine/threonine protein kinase involved in renal epithelial morphogenesis and in macrophage and granulocyte maturation; involved in nephrogenesis by stimulating renal epithelial cell migration and tubulogenesis; involved in angiogenesis through stimulation of endothelial cell proliferation, migration and vascular-like structure formation | Involved in embryonic development; expression more abundant in foetal brain, kidney, and liver tissues | (Li et al. 2005; Huang et al. 2016; Phung et al. 2022) |
| *STS* | Steroid sulfatase (arylsulfatase C) | 300747 | ND/XCI escapee | Membrane-bound microsomal enzyme catalyzes the conversion of sulfated steroid precursors, such as dehydroepiandrosterone sulfate (DHEA-S) and estrone sulfate to the free steroid | Overexpressed in placenta and syncytiotrophoblast; expression/activity increases in late pregnancy | (Dreyer et al. 2018; Phung et al. 2022) |
| *TUBGCP5* | Gamma-Tubulin Complex Component 5 | 608147 | Non-imprinted biallelically expressed gene/NA (chr 15) | Necessary for microtubule nucleation at the centrosome | Cytoplasmic expression in several tissues with low tissue specificity, including placenta; highly expressed in the most differentiated tissues | (Huang et al. 2021) |
| *XIST* | X Inactive Specific Transcript | 314670 | Non-imprinted biallelic/ subject to XCI | Essential for the initiation and spread of X-inactivation | Highly expressed in placental tissue from female foetuses | (Hoch et al. 2020; Phung et al. 2022) |
| *ZRSR2* | Zinc Finger CCCH-Type, RNA Binding Motif and Serine/Arginine-Rich 2 | 300028 | ND/XCI escapee (variable) | Essential splicing factor; associates with the U2 auxiliary factor heterodimer, which is required for the recognition of a functional 3' splice site in pre-mRNA splicing; required for assembly of the pre-spliceosome, a precursor to other spliceosomal complexes | Low tissue specificity of expression; expressed in the oocyte and the embryo needs the protein during early stages of development; essential for stem cell differentiation during gametogenesis in both males and females | (Gómez-Redondo et al. 2020; Phung et al. 2022) |

ND—no data; NA—not applicable

**Supplemental Table S2.** Gene expression in umbilical cord blood of groups with different degrees of X chromosome inactivation (XCI) skewing. Gene expression is presented as mean log_2_-transformed fold change (FC) calculated to mean expression level detected for samples with 50–59% XCI ratio. Positive values of log_2_FC mean upregulation of gene expression *vs*. the XCI 50–59% group, while negative values—downregulation. *P*-values were determined with *t*-test and were corrected for the false discovery rate (FDR) with Benjamini–Hochberg procedure (adjusted *p*-values—adj *p*). Significant *P*-values are presented in red; no adjusted *P*-values were smaller than the assumed FDR = 0.1.

| **Gene** | **XCI 60–69%** | | | **XCI 70–79%** | | |
| --- | --- | --- | --- | --- | --- | --- |
|  | **Log_2_ (FC)** | ***p*** | **adj *p*** | **Log_2_ (FC)** | ***p*** | **adj *p*** |
| *ABCA1* | 0.44 | 0.277 | 0.719 | −3.04 | 0.716 | 0.782 |
| *AR* | 1.99 | 0.308 | 0.719 | −1.65 | 0.920 | 0.782 |
| *BID* | −1.18 | 0.794 | 0.782 | −4.80 | 0.590 | 0.782 |
| *BLCAP* | 0.47 | 0.158 | 0.719 | −3.47 | 0.634 | 0.782 |
| *CD99* | −0.54 | 0.654 | 0.782 | −4.37 | 0.544 | 0.782 |
| *CDKN1C* | 0.68 | 0.316 | 0.719 | −4.62 | 0.758 | 0.782 |
| *H19* | 4.48 | 0.031 | 0.719 | −0.93 | 0.929 | 0.782 |
| *IGF2* | 1.53 | 0.241 | 0.719 | −0.04 | 0.808 | 0.782 |
| *KDM5C* | −1.39 | 0.443 | 0.782 | −7.00 | 0.738 | 0.782 |
| *MEG3* | −0.07 | 0.512 | 0.782 | −2.69 | 0.789 | 0.782 |
| *MEST* | 1.27 | 0.231 | 0.719 | −3.59 | 0.537 | 0.782 |
| *NAP1L5* | 2.04 | 0.111 | 0.719 | −1.83 | 0.752 | 0.782 |
| *JPX* | 0.00 | 0.386 | 0.741 | −4.42 | 0.618 | 0.782 |
| *NDN* | 0.31 | 0.314 | 0.719 | −4.81 | 0.791 | 0.782 |
| *P2RY8* | 2.16 | 0.091 | 0.719 | −2.85 | 0.444 | 0.782 |
| *STS* | 0.65 | 0.359 | 0.741 | −3.33 | 0.522 | 0.782 |
| *XIST* | 0.44 | 0.277 | 0.719 | −3.04 | 0.716 | 0.782 |

**Supplemental Table S3.** Gene expression in placental tissue of groups with different degrees of X chromosome inactivation (XCI) skewing. Gene expression is presented as mean log_2_-transformed fold change (FC) calculated to mean expression level detected for samples with 50–59% XCI ratio. Positive values of log_2_FC mean upregulation of gene expression *vs*. the XCI 50–59% group, while negative values—downregulation. *P*-values were determined with *t*-test and were corrected for the false discovery rate (FDR) with Benjamini–Hochberg procedure (adjusted *P*-values—adj *p*). Significant *P*-values are presented in red while adjusted *P*-values smaller than the assumed FDR = 0.1 are presented in green.

| **Gene** | **XCI 60–69%** | | | **XCI 70–79%** | | | **XCI 80–89%** | | | **XCI 90–100%** | | | |
| --- | --- | --- | --- | --- | --- | --- | --- | --- | --- | --- | --- | --- | --- |
|  | **Log_2_FC** | ***p*** | **adj *p*** | **Log_2_FC** | ***p*** | **adj *p*** | **Log_2_FC** | ***p*** | **adj *p*** | **Log_2_FC** | ***p*** | **adj *p*** |  |
| *ABCA1* | 0.09 | 0.598 | 0.716 | −1.13 | 0.282 | 0.530 | −1.36 | 0.139 | 0.242 | −2.58 | 0.171 | 0.324 |  |
| *ACE2* | −0.36 | 0.655 | 0.716 | 0.55 | 0.081 | 0.215 | 0.21 | 0.364 | 0.455 | 1.42 | 0.269 | 0.471 |  |
| *AR* | −0.70 | 0.900 | 0.900 | −0.99 | 0.997 | 0.997 | −2.90 | 0.088 | 0.192 | 0.40 | 0.587 | 0.802 |  |
| *BID* | −1.26 | 0.807 | 0.856 | −1.80 | 0.370 | 0.579 | −2.10 | 0.232 | 0.326 | −3.62 | 0.313 | 0.497 |  |
| *BLCAP* | 0.98 | 0.135 | 0.449 | 0.28 | 0.288 | 0.530 | 0.36 | 0.286 | 0.385 | 0.06 | 0.733 | 0.802 |  |
| *CD44* | 4.65 | 0.056 | 0.429 | 4.26 | 0.017 | 0.108 | 7.78 | 0.019 | 0.080 | 13.38 | 0.001 | 0.013 |  |
| *CD99* | −0.29 | 0.606 | 0.716 | −0.26 | 0.447 | 0.579 | 0.57 | 0.231 | 0.326 | −0.03 | 0.693 | 0.802 |  |
| *CDKN1C* | 0.65 | 0.170 | 0.449 | 0.47 | 0.262 | 0.530 | 0.76 | 0.191 | 0.304 | 0.20 | 0.713 | 0.802 |  |
| *DLK1* | 2.04 | 0.361 | 0.518 | 1.55 | 0.230 | 0.504 | 3.37 | 0.034 | 0.090 | 4.76 | 0.003 | 0.020 |  |
| *ERP27* | 1.31 | 0.205 | 0.449 | 2.48 | 0.039 | 0.132 | 5.09 | 0.021 | 0.080 | 8.60 | 0.014 | 0.060 |  |
| *GRB10* | 1.99 | 0.230 | 0.449 | 2.46 | 0.027 | 0.109 | 4.58 | 0.012 | 0.080 | 6.87 | 0.019 | 0.071 |  |
| *GYG2* | 1.36 | 0.266 | 0.485 | 0.97 | 0.095 | 0.221 | 1.27 | 0.118 | 0.229 | 2.05 | 0.108 | 0.251 |  |
| *H19* | 0.61 | 0.300 | 0.493 | −0.36 | 0.947 | 0.975 | −0.03 | 0.792 | 0.792 | −2.25 | 0.151 | 0.310 |  |
| *HECW2* | 3.34 | 0.152 | 0.449 | 3.46 | 0.016 | 0.108 | 5.29 | 0.023 | 0.080 | 6.45 | 0.003 | 0.019 |  |
| *IGF2* | 0.01 | 0.618 | 0.716 | 0.24 | 0.313 | 0.547 | 0.92 | 0.145 | 0.242 | 0.83 | 0.380 | 0.554 |  |
| *KDM5C* | 2.20 | 0.048 | 0.429 | 1.25 | 0.043 | 0.132 | 2.39 | 0.048 | 0.113 | 3.11 | 0.135 | 0.296 |  |
| *KDM6A* | 2.93 | 0.074 | 0.429 | 3.33 | 0.010 | 0.108 | 5.27 | 0.009 | 0.080 | 7.52 | 0.012 | 0.059 |  |
| *MEG3* | 1.01 | 0.230 | 0.449 | −1.00 | 0.888 | 0.942 | 0.25 | 0.458 | 0.552 | −0.82 | 0.993 | 0.993 |  |
| *MEST* | −0.52 | 0.878 | 0.900 | −0.51 | 0.876 | 0.942 | −0.66 | 0.602 | 0.658 | −1.73 | 0.330 | 0.503 |  |
| *NAP1L5* | 0.54 | 0.370 | 0.518 | 0.14 | 0.425 | 0.579 | −0.34 | 0.572 | 0.646 | −0.05 | 0.717 | 0.802 |  |
| *JPX* | 0.98 | 0.036 | 0.429 | 0.28 | 0.416 | 0.579 | 0.85 | 0.113 | 0.229 | 0.79 | 0.286 | 0.477 |  |
| *NDN* | 0.59 | 0.277 | 0.485 | −1.23 | 0.799 | 0.932 | −1.28 | 0.754 | 0.776 | −1.96 | 0.654 | 0.802 |  |
| *NNAT* | 0.76 | 0.483 | 0.627 | 0.55 | 0.404 | 0.579 | 1.84 | 0.031 | 0.090 | 2.84 | 0.055 | 0.160 |  |
| *OCA2* | 2.54 | 0.123 | 0.449 | 0.39 | 0.513 | 0.641 | 1.87 | 0.125 | 0.230 | 2.67 | 0.176 | 0.324 |  |
| *P2RY8* | 0.00 | 0.450 | 0.606 | −1.98 | 0.406 | 0.579 | −1.71 | 0.540 | 0.630 | −2.01 | 0.696 | 0.802 |  |
| *PAX8* | −6.11 | 0.310 | 0.493 | −4.26 | 0.436 | 0.579 | 0.42 | 0.233 | 0.326 | −1.93 | 0.941 | 0.969 |  |
| *PHLDA2* | 5.89 | 0.068 | 0.429 | 5.13 | 0.021 | 0.108 | 11.67 | 0.007 | 0.080 | 18.36 | 0.000 | 0.006 |  |
| *PLAGL1* | 3.67 | 0.103 | 0.449 | 3.57 | 0.022 | 0.108 | 6.32 | 0.007 | 0.080 | 8.88 | 0.020 | 0.071 |  |
| *PLCXD1* | 0.15 | 0.336 | 0.511 | −0.55 | 0.669 | 0.807 | −0.39 | 0.663 | 0.704 | 0.04 | 0.670 | 0.802 |  |
| *PNPLA4* | 1.53 | 0.185 | 0.449 | 2.73 | 0.004 | 0.062 | 3.17 | 0.032 | 0.090 | 5.01 | 0.026 | 0.084 |  |
| *PRKX* | 1.67 | 0.231 | 0.449 | 1.69 | 0.045 | 0.132 | 3.49 | 0.021 | 0.080 | 4.17 | 0.096 | 0.240 |  |
| *STS* | −0.91 | 0.644 | 0.716 | −0.27 | 0.854 | 0.942 | −1.10 | 0.353 | 0.455 | −0.77 | 0.805 | 0.854 |  |
| *TUBGCP5* | 2.24 | 0.206 | 0.449 | 2.64 | 0.028 | 0.109 | 4.07 | 0.023 | 0.080 | 7.11 | 0.000 | 0.006 |  |
| *XIST* | 1.61 | 0.107 | 0.449 | 1.20 | 0.086 | 0.215 | 2.33 | 0.048 | 0.113 | 2.72 | 0.087 | 0.234 |  |
| *ZRSR2* | 4.63 | 0.032 | 0.429 | 4.33 | 0.002 | 0.053 | 6.50 | 0.019 | 0.080 | 11.22 | 0.002 | 0.016 |  |

**Supplemental References**

Alanen HI, Williamson RA, Howard MJ, et al (2006) ERp27, a New Non-catalytic Endoplasmic Reticulum-located Human Protein Disulfide Isomerase Family Member, Interacts with ERp57. Journal of Biological Chemistry 281:33727–33738. https://doi.org/10.1074/jbc.M604314200

Aykroyd BRL, Tunster SJ, Sferruzzi-Perri AN (2020) Igf2 deletion alters mouse placenta endocrine capacity in a sexually dimorphic manner. Journal of Endocrinology 246:93–108. https://doi.org/10.1530/JOE-20-0128

Baumann M, Körner M, Huang X, et al (2013) Placental ABCA1 and ABCG1 expression in gestational disease: Pre-eclampsia affects ABCA1 levels in syncytiotrophoblasts. Placenta 34:1079–1086. https://doi.org/10.1016/j.placenta.2013.06.309

Berko ER, Cho MT, Eng C, et al (2017) De novo missense variants in HECW2 are associated with neurodevelopmental delay and hypotonia. J Med Genet 54:84–86. https://doi.org/10.1136/jmedgenet-2016-103943

Berland S, Haukanes BI, Juliusson PB, Houge G (2022) Deep exploration of a CDKN1C mutation causing a mixture of Beckwith-Wiedemann and IMAGe syndromes revealed a novel transcript associated with developmental delay. J Med Genet 59:155–164. https://doi.org/10.1136/JMEDGENET-2020-107401

Braun AE, Mitchel OR, Gonzalez TL, et al (2022) Sex at the interface: the origin and impact of sex differences in the developing human placenta. Biol Sex Differ 13:. https://doi.org/10.1186/S13293-022-00459-7

Chi Y-I, Stodola TJ, De Assuncao TM, et al (2021) Molecular mechanics and dynamic simulations of well-known Kabuki syndrome-associated KDM6A variants reveal putative mechanisms of dysfunction. Orphanet J Rare Dis 16:66. https://doi.org/10.1186/s13023-021-01692-w

Cleaton MAM, Dent CL, Howard M, et al (2016) Fetus-derived DLK1 is required for maternal metabolic adaptations to pregnancy and is associated with fetal growth restriction. Nat Genet 48:1473–1480. https://doi.org/10.1038/ng.3699

Cotton AM, Ge B, Light N, et al (2013) Analysis of expressed SNPs identifies variable extents of expression from the human inactive X chromosome. Genome Biol 14:1–17. https://doi.org/10.1186/GB-2013-14-11-R122/FIGURES/5

Davey RA, Grossmann M (2016) Androgen Receptor Structure, Function and Biology: From Bench to Bedside. Clin Biochem Rev 37:3–15

Deyssenroth MA, Li Q, Escudero C, et al (2020) Differences in Placental Imprinted Gene Expression across Preeclamptic and Non-Preeclamptic Pregnancies. Genes (Basel) 11:1146. https://doi.org/10.3390/genes11101146

Dreyer F-E, Abdulrahman GO, Waring G, Hinshaw K (2018) Placental steroid sulphatase deficiency: an approach to antenatal care and delivery. Ann Saudi Med 38:445–449. https://doi.org/10.5144/0256-4947.2018.445

Fagerberg L, Hallström BM, Oksvold P, et al (2014) Analysis of the Human Tissue-specific Expression by Genome-wide Integration of Transcriptomics and Antibody-based Proteomics. Molecular & Cellular Proteomics 13:397–406. https://doi.org/10.1074/mcp.M113.035600

Ferretti E, Arturi F, Mattei T, et al (2005) Expression, Regulation, and Function of Paired-Box Gene 8 in the Human Placenta and Placental Cancer Cell Lines. Endocrinology 146:4009–4015. https://doi.org/10.1210/en.2005-0084

Gabory A, Ripoche M-A, Yoshimizu T, Dandolo L (2006) The H19 gene: regulation and function of a non-coding RNA. Cytogenet Genome Res 113:188–193. https://doi.org/10.1159/000090831

Gao Y-Y, Chen L, Wang T, et al (2016) Oocyte aging-induced Neuronatin (NNAT) hypermethylation affects oocyte quality by impairing glucose transport in porcine. Sci Rep 6:36008. https://doi.org/10.1038/srep36008

Gómez-Redondo I, Ramos-Ibeas P, Pericuesta E, et al (2020) Minor Splicing Factors Zrsr1 and Zrsr2 Are Essential for Early Embryo Development and 2-Cell-Like Conversion. Int J Mol Sci 21:4115. https://doi.org/10.3390/ijms21114115

Gonzalez TL, Sun T, Koeppel AF, et al (2018) Sex differences in the late first trimester human placenta transcriptome. Biol Sex Differ 9:4. https://doi.org/10.1186/s13293-018-0165-y

Gu T, Su X, Zhou Q, et al (2012) Molecular Characterization of the Neuronatin Gene in the Porcine Placenta. PLoS One 7:e43325. https://doi.org/10.1371/journal.pone.0043325

Guo N, Zheng D, Sun J, et al (2021) NAP1L5 Promotes Nucleolar Hypertrophy and Is Required for Translation Activation During Cardiomyocyte Hypertrophy. Front Cardiovasc Med 0:1956. https://doi.org/10.3389/FCVM.2021.791501

Hamed M, Ismael S, Paulsen M, Helms V (2012) Cellular Functions of Genetically Imprinted Genes in Human and Mouse as Annotated in the Gene Ontology. PLoS One 7:50285. https://doi.org/10.1371/journal.pone.0050285

Harris LK, Crocker IP, Baker PN, et al (2011) IGF2 Actions on Trophoblast in Human Placenta Are Regulated by the Insulin-Like Growth Factor 2 Receptor, Which Can Function as Both a Signaling and Clearance Receptor. Biol Reprod 84:440. https://doi.org/10.1095/BIOLREPROD.110.088195

Hecht JL, Quade B, Deshpande V, et al (2020) SARS-CoV-2 can infect the placenta and is not associated with specific placental histopathology: a series of 19 placentas from COVID-19-positive mothers. Modern Pathology 33:2092–2103. https://doi.org/10.1038/s41379-020-0639-4

Hermansson M, Hänninen S, Hokynar K, Somerharju P (2016) The PNPLA-family phospholipases involved in glycerophospholipid homeostasis of HeLa cells. Biochimica et Biophysica Acta (BBA) - Molecular and Cell Biology of Lipids 1861:1058–1065. https://doi.org/10.1016/j.bbalip.2016.06.007

Hivert M-F, Cardenas A, Allard C, et al (2020) Interplay of Placental DNA Methylation and Maternal Insulin Sensitivity in Pregnancy. Diabetes 69:484–492. https://doi.org/10.2337/db19-0798

Hoch D, Novakovic B, Cvitic S, et al (2020) Sex matters: XIST and DDX3Y gene expression as a tool to determine fetal sex in human first trimester placenta. Placenta 97:68–70. https://doi.org/10.1016/j.placenta.2020.06.016

Hsu T-Y, Lan K-C, Tsai C-C, et al (2009) Expression of Androgen Receptor in Human Placentas from Normal and Preeclamptic Pregnancies. Taiwan J Obstet Gynecol 48:262–267. https://doi.org/10.1016/S1028-4559(09)60301-6

Huang S, Li Q, Alberts I, Li X (2016) PRKX, a Novel cAMP-Dependent Protein Kinase Member, Plays an Important Role in Development. J Cell Biochem 117:566–573. https://doi.org/10.1002/jcb.25304

Huang X, Chen J, Hu W, et al (2021) A report on seven fetal cases associated with 15q11‐q13 microdeletion and microduplication. Mol Genet Genomic Med 9:e1605. https://doi.org/10.1002/mgg3.1605

Imagawa E, Osaka H, Yamashita A, et al (2014) A hemizygous GYG2 mutation and Leigh syndrome: a possible link? Hum Genet 133:225–234. https://doi.org/10.1007/s00439-013-1372-6

Kappil MA, Green BB, Armstrong DA, et al (2015) Placental expression profile of imprinted genes impacts birth weight. Epigenetics 10:842–849. https://doi.org/10.1080/15592294.2015.1073881

Krajewska M, Mai JK, Zapata JM, et al (2002) Dynamics of expression of apoptosis-regulatory proteins Bid, Bcl-2, Bcl-X, Bax and Bak during development of murine nervous system. Cell Death Differ 9:145–157. https://doi.org/10.1038/sj.cdd.4400934

Lambertini L, Marsit CJ, Sharma P, et al (2012) Imprinted gene expression in fetal growth and development. Placenta 33:480–486. https://doi.org/10.1016/j.placenta.2012.03.001

Li W, Yu Z-X, Kotin RM (2005) Profiles of PrKX Expression in Developmental Mouse Embryo and Human Tissues. Journal of Histochemistry & Cytochemistry 53:1003–1009. https://doi.org/10.1369/jhc.4A6568.2005

Lu E, Wolfreys FD, Muppidi JR, et al (2019) S-Geranylgeranyl-L-glutathione is a ligand for human B cell-confinement receptor P2RY8. Nature 567:244–248. https://doi.org/10.1038/s41586-019-1003-z

Monnier P, Martinet C, Pontis J, et al (2013) H19 lncRNA controls gene expression of the Imprinted Gene Network by recruiting MBD1. Proceedings of the National Academy of Sciences 110:20693–20698. https://doi.org/10.1073/pnas.1310201110

Moore GE, Ishida M, Demetriou C, et al (2015) The role and interaction of imprinted genes in human fetal growth. Philosophical Transactions of the Royal Society B: Biological Sciences 370:20140074. https://doi.org/10.1098/rstb.2014.0074

Mukhopadhyay A, Ravikumar G, Dwarkanath P, et al (2015) Placental expression of the insulin receptor binding protein GRB10: Relation to human fetoplacental growth and fetal gender. Placenta 36:1225–1230. https://doi.org/10.1016/j.placenta.2015.09.006

Navarro-Cobos MJ, Balaton BP, Brown CJ (2020) Genes that escape from X-chromosome inactivation: Potential contributors to Klinefelter syndrome. https://doi.org/10.1002/ajmg.c.31800

Nikitina L, Wenger F, Baumann M, et al (2011) Expression and localization pattern of ABCA1 in diverse human placental primary cells and tissues. Placenta 32:420–430. https://doi.org/10.1016/j.placenta.2011.03.003

Patrat C, Ouimette J-F, Rougeulle C (2020) X chromosome inactivation in human development. Development 147:dev183095. https://doi.org/10.1242/dev.183095

Phung TN, Olney KC, Pinto BJ, et al (2022) X chromosome inactivation in the human placenta is patchy and distinct from adult tissues. Human Genetics and Genomics Advances 3:100121. https://doi.org/10.1016/j.xhgg.2022.100121

Plasschaert RN, Bartolomei MS (2015) Tissue-specific regulation and function of Grb10 during growth and neuronal commitment. Proceedings of the National Academy of Sciences 112:6841–6847. https://doi.org/10.1073/pnas.1411254111

Renshaw SA, Dempsey CE, Barnes FA, et al (2004) Three Novel Bid Proteins Generated by Alternative Splicing of the Human Bid Gene. Journal of Biological Chemistry 279:2846–2855. https://doi.org/10.1074/jbc.M309769200

Starks RR, Abu Alhasan R, Kaur H, et al (2020) Transcription Factor PLAGL1 Is Associated with Angiogenic Gene Expression in the Placenta. Int J Mol Sci 21:8317. https://doi.org/10.3390/ijms21218317

Tunster SJ, Tycko B, John RM (2010) The Imprinted Phlda2 Gene Regulates Extraembryonic Energy Stores. Mol Cell Biol 30:295–306. https://doi.org/10.1128/MCB.00662-09

Vega-Benedetti AF, Saucedo C, Zavattari P, et al (2017) PLAGL1: an important player in diverse pathological processes. J Appl Genet 58:71–78. https://doi.org/10.1007/s13353-016-0355-4

Walters KA, Middleton LJ, Joseph SR, et al (2012) Targeted Loss of Androgen Receptor Signaling in Murine Granulosa Cells of Preantral and Antral Follicles Causes Female Subfertility1. Biol Reprod 87:151–152. https://doi.org/10.1095/biolreprod.112.102012

Wood AJ, Roberts RG, Monk D, et al (2007) A Screen for Retrotransposed Imprinted Genes Reveals an Association between X Chromosome Homology and Maternal Germ-Line Methylation. PLoS Genet 3:e20. https://doi.org/10.1371/JOURNAL.PGEN.0030020

Yonekura S, Ohata M, Tsuchiya M, et al (2019) Peg1/Mest, an imprinted gene, is involved in mammary gland maturation. J Cell Physiol 234:1080–1087. https://doi.org/10.1002/jcp.27219

Yuen R, Gray SG, Morison I, Monk D (2016) Epigenetic Characterization of CDKN1C in Placenta Samples from Non-syndromic Intrauterine Growth Restriction. https://doi.org/10.3389/fgene.2016.00062

Zhang X, Zhou Y, Mehta KR, et al (2003) A Pituitary-Derived MEG3 Isoform Functions as a Growth Suppressor in Tumor Cells. J Clin Endocrinol Metab 88:5119–5126. https://doi.org/10.1210/jc.2003-030222
