# Supplementary material for: X-chromosome inactivation patterns depend on age and tissue but not conception method in humans
Source: Chromosome Res. 2023 Jan 25;31(1):4. doi: 10.1007/s10577-023-09717-9 (PMC9877087; doi:10.1007/s10577-023-09717-9)
Supplement: Supplementary file 3 — Supplementary file3 (DOCX 657 KB) [file 10577_2023_9717_MOESM3_ESM.docx]

**X chromosome inactivation patterns depend on age and tissue but not conception method in humans**

Patrycja Juchniewicz^1^, Anna Kloska^1*^, Karolina Portalska^2^, Joanna Jakóbkiewicz-Banecka^1^, Grzegorz Węgrzyn^2^, Joanna Liss^3,1^, Piotr Głodek^3^, Stefan Tukaj^2^, Ewa Piotrowska^2*^

^1^ Department of Medical Biology and Genetics, Faculty of Biology, University of Gdańsk, Gdańsk, Poland

^2^ Department of Molecular Biology, Faculty of Biology, University of Gdańsk, Gdańsk, Poland

^3^ Research and Development Center, INVICTA, Sopot, Poland

^*^**Correspondence:**

Ewa Piotrowska, University of Gdańsk, Department of Molecular Biology, Wita Stwosza 59, 80-308 Gdańsk, Poland. Tel. +48 58 523 6040; e-mail: ewa.piotrowska@ug.edu.pl

Anna Kloska, University of Gdańsk, Department of Medical Biology and Genetics, Wita Stwosza 59, 80-308 Gdańsk, Poland. Tel. +48 58 523 6044; e-mail: anna.kloska@ug.edu.pl

**Supplemental Figures**

**
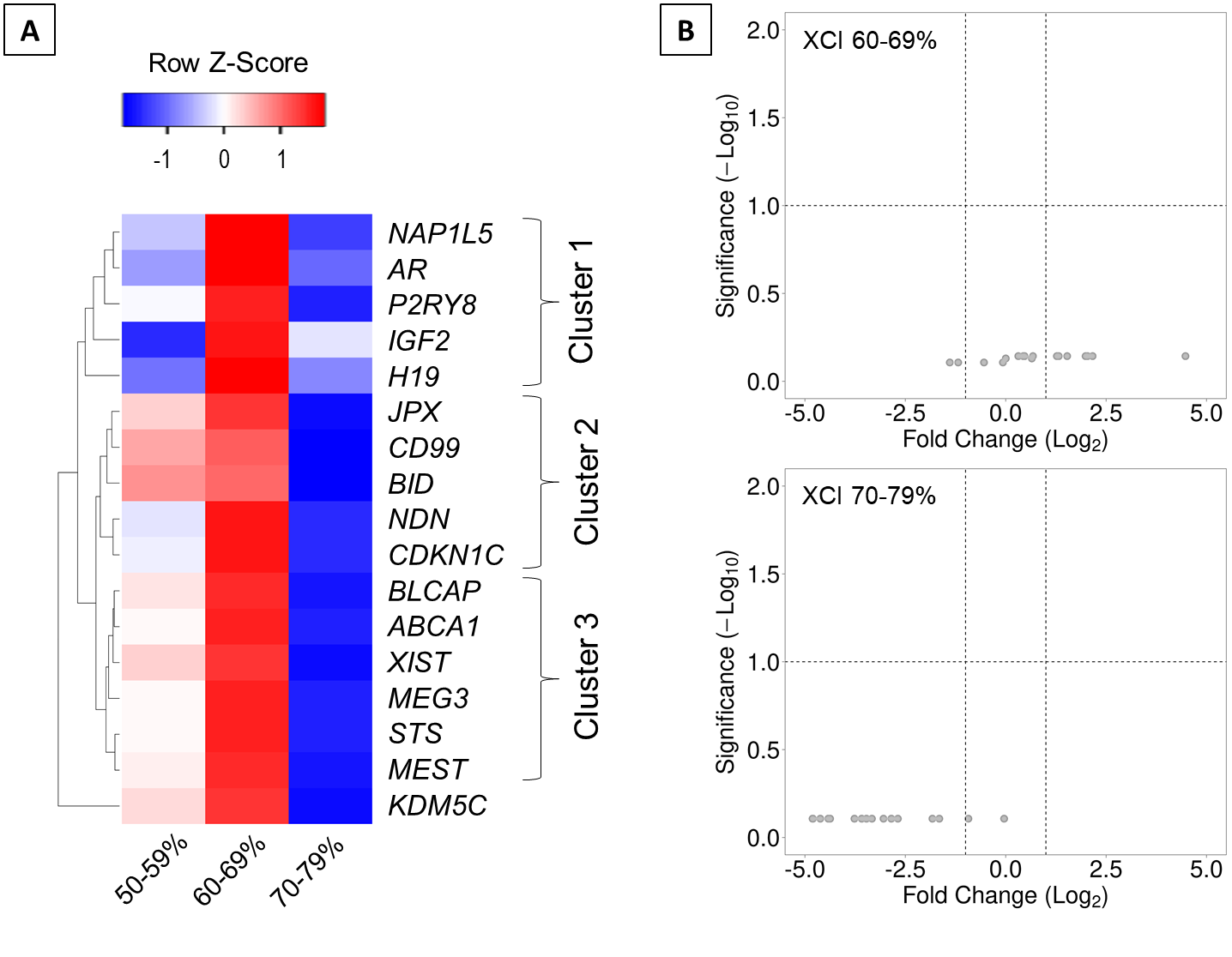
**

Supplemental **Fig. S1** Gene expression profile of umbilical cord blood of different X chromosome inactivation (XCI) groups. (A) Heat map showing gene expression fold change in cord blood; data are presented as row z-score. Hierarchical clustering is based on mean log_2_-transformed fold change values with the average linkage as the clustering method and the Euclidean distance measurement method. (B) Volcano plots presenting gene expression changes in groups with different XCI statuses. Gene expression fold-change threshold is set to log_2_FC = |1.0| and significance threshold is set to −log_10_(adjusted *P*-value) = 1.0. The grey dots represent unchanged expression according to the set thresholds.


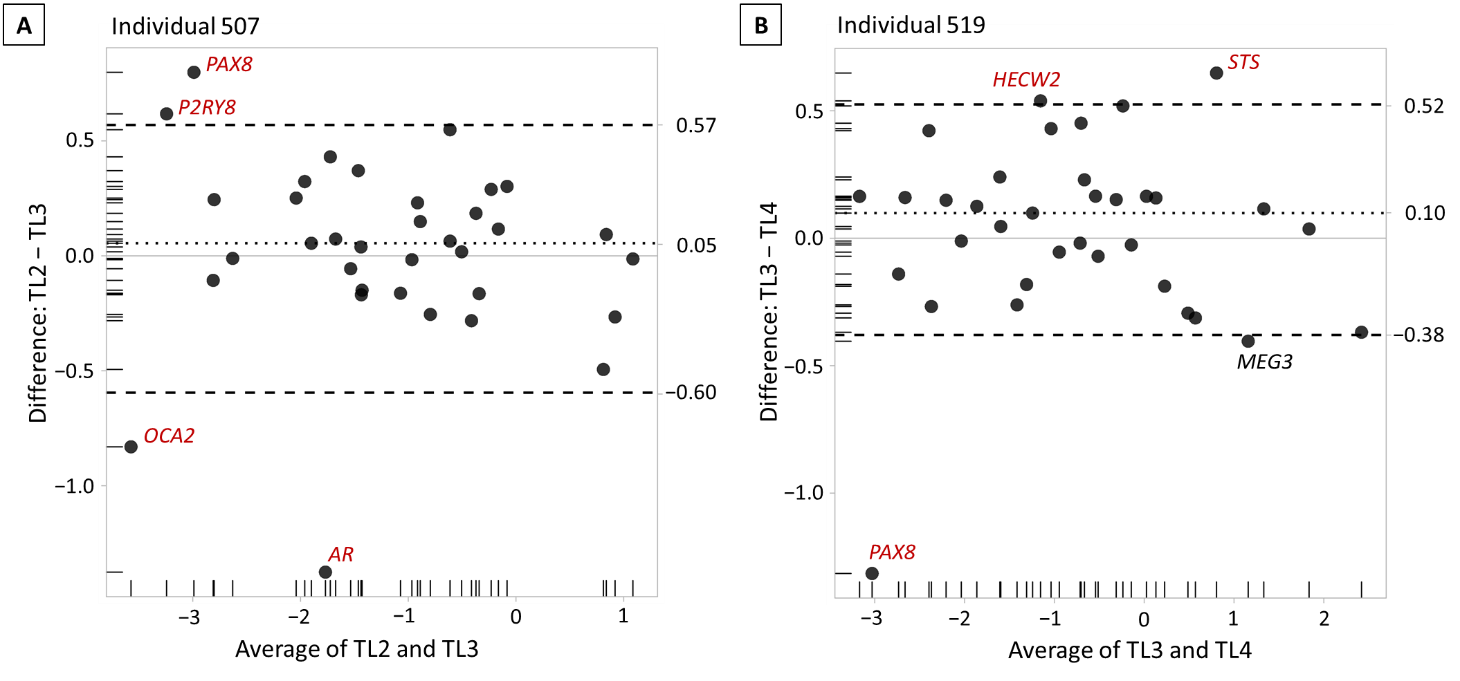


Supplemental **Fig. S2** Bland–Altman plots for estimation of agreement between relative expression values obtained for pairs of placental tissue samples with inverted, skewed XCI ratios of individual 507 (**A**) and 519 (**B**). Log_10_-transformed, relative expression (RE) data were used for calculations and graphs were constructed by plotting the difference between the two RE values obtained for corresponding placental tissue samples (TL2 and TL3 of individual 507; TL3 and TL4 of individual 519) (Y-axis) against the average of the two RE values obtained for both samples (X-axis). The dotted line indicates the average difference and the dashed lines indicate the limits of agreement (determined with the non-parametric method using BA-plotteR web tool (Goedhart and Rishniw 2021)). Each point represents data for a specific gene from the gene expression panel used in this study. If the expression level for a gene is similar in both samples, the corresponding point is scattered between the determined upper and lower limits of agreement; points scattered outside the limits of agreement show inconsistent expression levels in both examined samples (gene names labelled in red.


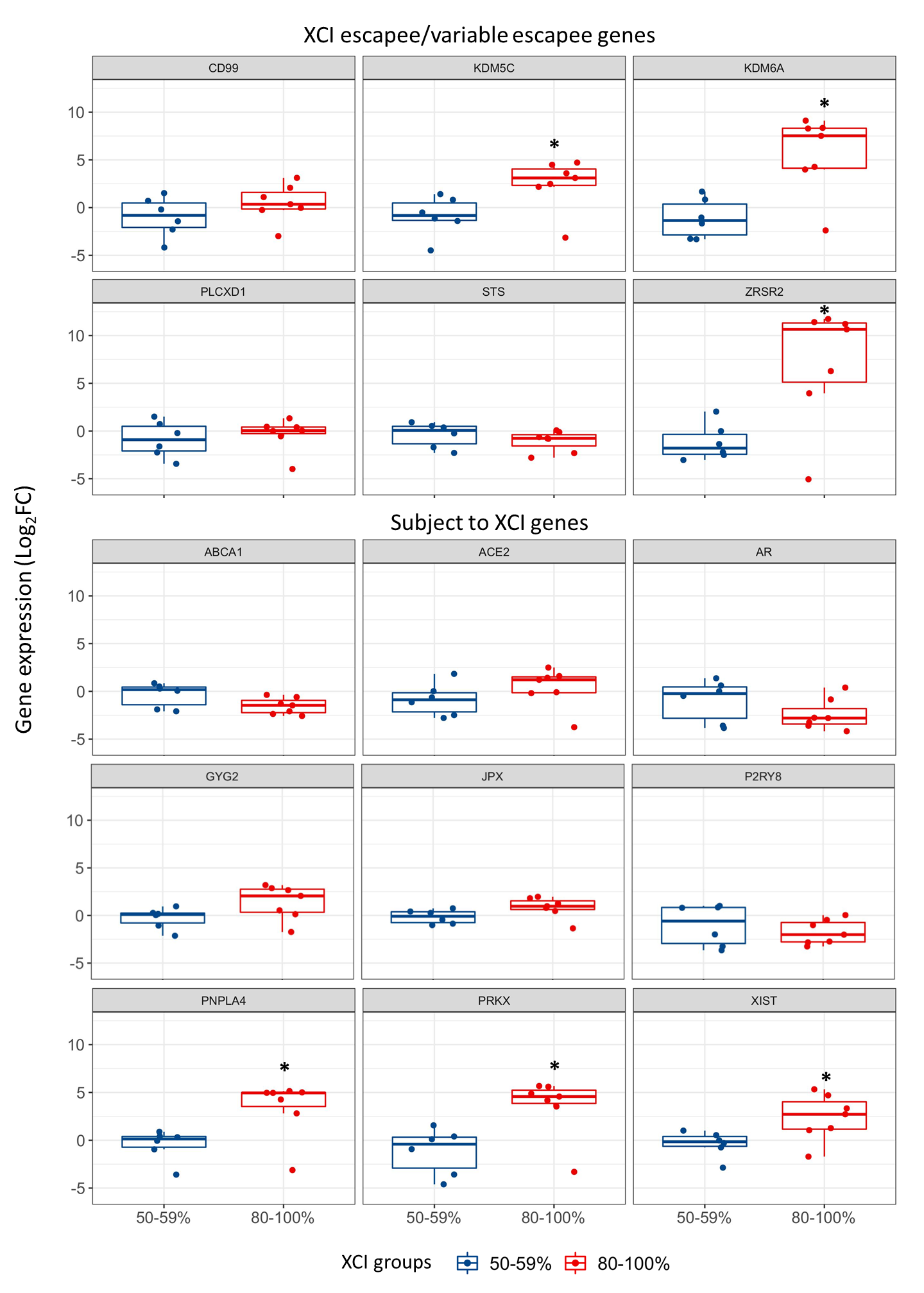


Supplemental **Fig. S3** Gene expression in placental tissue for X-linked genes. Genes are classified as XCI escapee, variable XCI escapee gene or genes subjected to XCI. Box plots show gene expression determined for a group with the most random XCI pattern (XCI of 50-59%) and a group with the most skewed XCI pattern (XCI of 80-100%; pooled). The box shows lower and upper quartiles and whiskers—minimum and maximum values of the data; the horizontal line inside the box represents median and dots—individual samples; the points located outside the box represent outliers. The black asterisk (*) denotes a significant (*P*< 0.05) difference compared to the XCI 50–59% group determined with the *t*-test.
